# Plasma Metabolites Associated with CKD Stage in Autosomal Dominant Tubulointerstitial Kidney Disease

**DOI:** 10.34067/KID.0000001029

**Published:** 2025-11-14

**Authors:** Dita Mušálková, Martin Radina, Kendrah Kidd, Hana Hartmannová, Helena Trešlová, Kateřina Hodaňová, Petr Vyleťal, Alena Vrbacká, Miroslav Votruba, Antonio Sanchez, Lauren Martin, Abbigail Taylor, Alice Kim, Lucie Rudl Kulhavá, Jiří Hricko, Tomáš Čajka, Martina Živná, Anthony J. Bleyer, Stanislav Kmoch

**Affiliations:** 1 Research Unit for Rare Diseases, Department of Pediatrics and Inherited Metabolic Disorders, First Faculty of Medicine, Charles University, Prague, Czech Republic; 2 Section on Nephrology, Wake Forest University School of Medicine, Winston-Salem, North Carolina; 3 Institute of Physiology of the Czech Academy of Sciences, Prague, Czech Republic

**Keywords:** CKD, metabolomics, tubulointerstitial disease

## Abstract

**Key Points:**

- This is the first large-scale metabolomic study in genetically confirmed autosomal dominant tubulointerstitial kidney disease (ADTKD), providing a new resource for rare kidney diseases.
- ADTKD-*UMOD* and ADTKD-*MUC1* are metabolically indistinguishable across stages, supporting the development of unified monitoring strategies.
- The plasma kynurenine-to-tryptophan ratio increases with CKD progression, supporting its use as a noninvasive marker of inflammation in ADTKD.

**Background:**

Metabolomic profiling has not yet been performed in autosomal dominant tubulointerstitial kidney disease (ADTKD) due to *UMOD* or *MUC1* mutations and could provide valuable insights into the pathophysiology of these conditions and identify the biomarkers of disease activity.

**Methods:**

Untargeted metabolomic analysis of plasma samples was performed on a cohort comprising 40 controls, 51 individuals with ADTKD-*UMOD*, and 49 individuals with ADTKD-*MUC1* with CKD stages ranging from 1 to 4.

**Results:**

Principal component analysis and hierarchical clustering revealed that the metabolic profiles of controls and ADTKD-*UMOD* and ADTKD-*MUC1* patients with CKD stages 1 and 2 were similar. The metabolome was also similar between patients with ADTKD-*UMOD* and ADTKD-*MUC1* at each stage of CKD. Compared with stage 2 CKD, stage 3 CKD was characterized by an increased kynurenine-to-tryptophan ratio, indicating activation of indoleamine 2,3-dioxygenase, an inflammation-induced and rate-limiting enzyme of tryptophan metabolism, and increased levels of pseudouridine, 3-indoxylsulfate, *N*-formylmethione, *N*-acetylated amino acids, acylcarnitines, and several other metabolites. In total, 121 metabolites were identified as significantly altered in patients in stage 4 compared with controls. Enrichment analysis of this set revealed that the most significant alterations were in the biosynthesis of arginine and branched-chain amino acids, carnitine synthesis, transfer RNA metabolism, tryptophan catabolism, urea cycle, metabolism of amino acids, glucose homeostasis, and solute carrier-mediated transmembrane transport.

**Conclusions:**

Patients with ADTKD-*UMOD* and ADTKD-*MUC1* had similar metabolomic profiles across CKD stages. Although ADTKD is a tubulointerstitial kidney disease rather than glomerular, the effects on the metabolomic pathways appear comparable with those of other forms of CKD. The kynurenine-to-tryptophan ratio appears to be a promising biomarker of ADTKD progression and will require additional study.

## Visual Abstract

**Figure:**
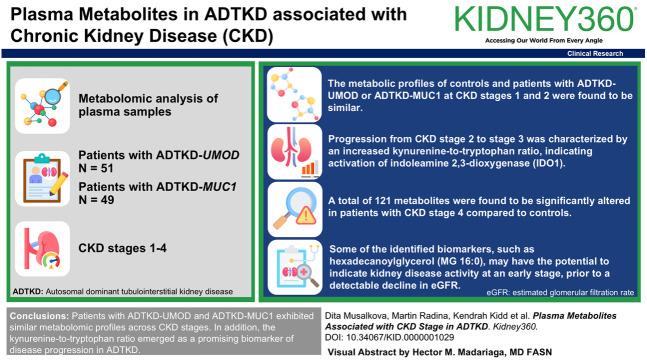

## Introduction

Autosomal dominant tubulointerstitial kidney disease (ADTKD) is an increasingly recognized condition with three primary characteristics: autosomal dominant inheritance, bland urinary sediment (absence of hematuria and proteinuria), and CKD leading to kidney failure (need for KRT or kidney transplantation) between 20 and 80 years, with a mean age of kidney failure of approximately 45 years.^1,2^

The two main causes of ADTKD are mutations in the *UMOD*^3^ and *MUC1*^4^ genes that encode uromodulin^5^ and mucin-1^6^ proteins, respectively. These diseases^7^ are caused by the intracellular deposition of the corresponding mutant proteins, which leads to endoplasmic reticulum stress and accelerated tubular cell damage.^8–11^

Although there are several potential therapies for each disease in sight,^8,10,12–14^ we cannot plan an interventional trial at this time because of the lack of biomarkers of disease activity or surrogate end points of disease progression. In both disease types, the marked variation in the rate of decline of the GFR^2,15,16^ is an obstacle to the performance of prospective interventional trials because it is difficult to stratify participants and predict how many individuals will reach trial end points. In addition, unlike glomerular diseases, proteinuria cannot be used as a surrogate end point.

As a first step in identifying biomarkers of CKD progression in these two genetic disorders, we performed untargeted metabolomic and lipidomic analyses of plasma samples from 40 control individuals from families with ADTKD-*UMOD* and ADTKD-*MUC1*, 51 individuals with ADTKD-*UMOD*, and 49 individuals with ADTKD-*MUC1* across CKD stages 1 to 4, assessing the association of metabolites with disease progression. In parallel, we explored dysregulated metabolic pathways that could provide insight into underlying disease mechanisms and their relationship to kidney function decline, whether specific to ADTKD-*UMOD* or ADTKD-*MUC1* individually and/or potentially relevant to CKD more broadly.

## Methods

### Study Participants

This investigation was approved by the Institutional Review Boards of Wake Forest School of Medicine and the First Faculty of Medicine, Charles University. Written informed consent was obtained from all participants. Plasma samples were obtained from patients and their genetically unaffected relatives recruited by the Rare Inherited Kidney Disease Team of Wake Forest University School of Medicine, Winston-Salem, NC,^17^ and stored at −80°C. The genetic diagnosis of ADTKD-*UMOD* and ADTKD-*MUC1* was established as previously described.^18–20^ CKD staging was performed using the CKD-Epidemiology Collaboration equation.

### Metabolite Analysis

Samples were processed, and liquid chromatography–mass spectrometry (LC-MS) analysis was performed, as previously described.^21^ Instrumental files generated from LC-MS analyses were processed using MS-DIAL version 4.9.221218 software.^22^ Methodology and statistical analyses are detailed in Supplemental Methods. Pathway analysis was performed in MetaboAnalyst version 6.0.^23^

## Results

### Demographic and Clinical Characteristics of the Study Participants

**Figure 1:**
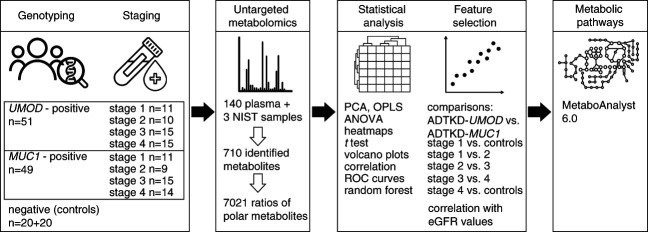
**Overview of the project.** ADTKD, Autosomal dominant tubulointerstitial kidney disease; OPLS, orthogonal partial least squares analysis; PCA, principal component analysis; ROC, receiver operating characteristic.

Metabolomic analysis was performed on 143 plasma samples. Patient plasma samples (*n*=100) were obtained from 11, ten, 15, and 15 individuals with ADTKD-*UMOD* and 11, nine, 15, and 14 individuals with ADTKD-*MUC1* with CKD stages 1, 2, 3, and 4, respectively. Control plasma samples (*n*=40) were obtained from genetically unaffected relatives, with 20 coming from ADTKD-*UMOD* and 20 coming from ADTKD-*MUC1* families (referred to as *UMOD* and *MUC1* family controls). Standard reference plasma samples National Institute of Standards and Technology SRM 1950 (*n*=3) were obtained from the National Institute of Standards and Technology. Characteristics of the study population are shown in Table 1. The project is summarized in Figure 1.

**Table 1:** Characteristics of the study population

Characteristic	ADTKD-*UMOD*	ADTKD-*MUC1*	*P*-Value (*UMOD* vs *MUC1*)	Genetically Unaffected *UMOD* Family	Genetically Unaffected *MUC1* Family	*P* Value Genetically Unaffected
Individuals, n	51	49	n/a	20	20	n/a
Families, n	43	36	n/a	20	14	n/a
Male sex, n (%)	13 (26%)	13 (27%)	0.90448	9 (45%)	7 (35%)	0.6455
Age (yr), mean±SD	38.1±14.5	36.6±16.5	0.98258	42.6±16.5	39.6±17.3	0.578239
Presence of gout, n (%)	21 (41%)	2 (4%)	<0.00001	0	1(5%)	0.29372
BMI mean±SD	25.7±5.9	24.3±5.9	0.258583	26.7±4.9	26.1±3.3	0.97247
**Race**						
Asian or Pacific Islander, n (%)	2 (4%)	1 (2%)	0.58232	1(5%)	0	n/a
White, n (%)	49 (96%)	48 (98%)	0.58	19 (95%)	20 (100%)	0.3125
**Ethnicity**						
Hispanic or Latino, n (%)	1 (2%)	9 (18%)	0.00634	1 (5%)	3 (15%)	0.29372
Not Hispanic or Latino, n (%)	50 (98%)	40 (82%)	0.006	19 (95%)	17 (85%)	0.29
**Other comorbidities**						
Diabetes, n (%)	1 (2%)	1 (2%)	0.97606	0	0	n/a
Active cancer diagnosis, n (%)	0	0	n/a	0	0	n/a
Hyperlipidemia at risk of cardiovascular disease, n (%)	2 (4%)	1 (2%)	0.58323	1 (5%)	0	n/a
Current smoker, n (%)	3 (6%)	2 (4%)	0.6818	3 (15%)	1 (5%)	0.29372
**By eGFR range**						
eGFR >60, n (%)	21 (41%)	20 (41%)	0.9681	19 (95%)	20 (100%)	0.3125
Age mean±SD	33.6±14.8	28.1±11.6	0.192964	41.1±15.5	39.6±17.3	0.778392
Serum creatinine mean±SD	0.83±0.16	0.86±0.20	0.590624	0.84±0.21	0.89±0.20	0.89289
eGFR 30–60, n (%)	15 (29%)	15 (29%)	0.89656	1 (5%)	0	n/a
Age mean±SD	36.1±9.7	41.6±14.0	0.219469	71.22	—	n/a
Serum creatinine mean±SD	1.76±0.38	1.61±0.38	0.95341	1.44	—	n/a
eGFR <30, n (%)	15 (29%)	14 (29%)	0.92828	0	0	n/a
Age mean±SD	46.3±15.4	43.4±13.9	0.588431	—	—	n/a
Serum creatinine mean±SD	2.46±0.48	3.36±1.46	0.030525	—	—	n/a

ADTKD, autosomal dominant tubulointerstitial kidney disease; BMI, body mass index.

### Metabolomic Analysis

Combining results from four LC-MS platforms, we annotated 710 metabolites, mostly complex lipids, followed by polar metabolites, and exposome compounds (Supplemental Data 1). Creatinine levels obtained from LC-MS analysis correlated very highly with clinical laboratory measurements of serum creatinine (Spearman r=0.97, *P* < 2×10^−16^) and calculated eGFR values (Spearman r = −0.91, *P* < 2×10^−16^).

### Multivariate Analysis

To classify samples and identify the metabolites whose concentration changes contribute most to variation, we applied unsupervised principal component analysis (PCA) and supervised orthogonal partial least squares analysis (OPLS). As shown in Supplemental Figure 1, A–D, the samples separated according to the eGFR values, with the largest differences between controls and patients with CKD stage 4. The top 50 metabolites contributing most to the separation in PCA and the top 50 metabolites with the highest variable importance of projection identified in OPLS overlapped in both groups (Supplemental Figure 1, E and F). To visualize the contribution of these metabolites to the original classification of CKD stage and ADTKD genetic subtype, we applied hierarchical clustering analysis (Supplemental Figure 1G). The resulting clustering dendrograms revealed that controls and ADTKD-*UMOD* and ADTKD-*MUC1* patients with CKD stages 1 and 2 were all similar and overlapped. By contrast, plasma samples from CKD stages 3 and 4 formed clearly distinct clusters. In a few cases, we observed clustering of the patient samples with samples from adjacent CKD stages. Samples from the ADTKD-*UMOD* and ADTKD-*MUC1* groups clustered together, suggesting minimal or no differences in their metabolic profiles. Key metabolites driving the clustering of samples in relation to eGFR values included pseudouridine, creatinine, dimethylarginine, acetylthreonine, *N*-formylmethionine, *N*-acetylalanine, 1-methylhistidine, *N*-acetylserine, and urea (Supplemental Figure 1, E, F, and H).

### ANOVA Analysis

To identify metabolites whose concentrations varied between CKD stages and ADTKD genetic subtypes, we performed ANOVA analysis and Tukey honestly significant difference *post hoc* tests. This analysis identified 95 metabolites with significantly altered levels (Supplemental Data 2). Hierarchical clustering of the samples on the basis of the metabolites identified as significant by ANOVA showed results that were analogous to the previous hierarchical clustering analysis on the basis of the results of PCA and OPLS (Supplemental Figure 2). All of the top 50 contributors to the separation of samples from PCA analysis and the top 50 contributors with the highest variable importance of projection values from the OPLS model were found to be significant by ANOVA analysis. Vice versa, ANOVA-significant metabolites contributed most to the separation of samples in PCA and OPLS analysis and correlated with eGFR.

### Comparison of *UMOD* and *MUC1* Family Controls and Establishment of the Pooled Control Group

On the basis of ANOVA results, we first tested for and found no statistically significant differences between the 20 control samples from ADTKD-*UMOD* families and the 20 control samples from ADTKD-*MUC1* families. This justified merging the data from these two control groups into a single pooled control group (*n*=40), thereby increasing the statistical power for all of the subsequent univariate tests.

### Specific Biomarkers of ADTKD-*UMOD* or ADTKD-*MUC1*

**Figure 2:**
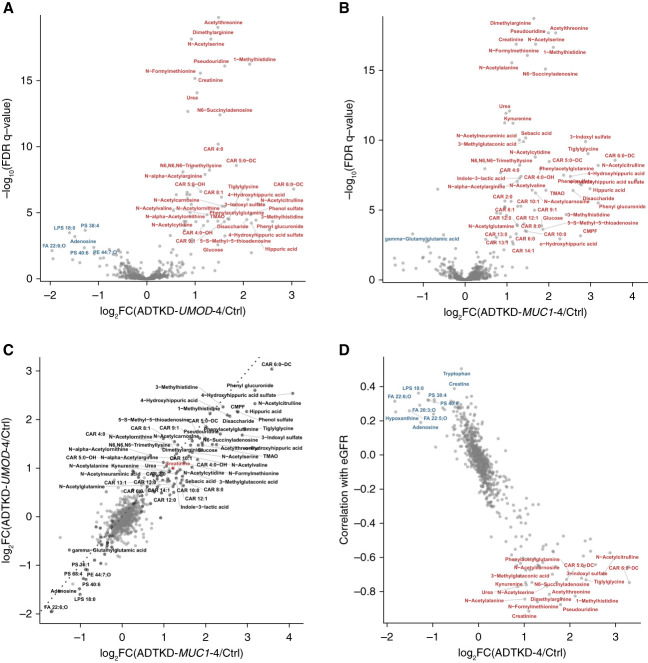
**Comparison of patients with ADTKD-*UMOD* and ADTKD-*MUC1* in stage 4 and control groups.** Volcano plots highlight the differences between patients with ADTKD-*UMOD* in stage 4 of the disease and controls (A) and patients with ADTKD-*MUC1* in stage 4 of the disease and controls (B). Names are shown only in metabolites with FDR-adjusted *P* value <0.01 and absolute value of log_2_ fold change >1. Metabolites in blue and red are present in lower levels and higher levels in the patients, respectively. (C) Log_2_ fold changes (shown in A and B) correlate very strongly (Pearson r=0.89, *P* < 2×10^−16^). Creatinine is highlighted by red color. (D) The relation between log_2_ fold changes (all patients in stage 4 versus controls) and Spearman correlation coefficients (correlation of the metabolite values with eGFR values across all samples). Ctrl, control; FC, fold change; FDR, false discovery rate.

One of the goals of this study was to determine whether any metabolites could biochemically distinguish ADTKD-*UMOD* from ADTKD-*MUC1*. To this end, we compared age- and sex-matched groups and, in accordance with ANOVA results, did not identify any metabolites that significantly differentiated ADTKD-*UMOD* and ADTKD-*MUC1* groups in CKD stage 4 (Figure 2) or in other CKD stages (data not shown). Thus, as in the control group, this allowed us to pool data from ADTKD-*UMOD* and ADTKD-*MUC1* groups for individual CKD stages for subsequent statistical analyses, with 22 participants in ADTKD-1 (ADTKD stage 1), 19 participants in ADTKD-2, 30 participants in ADTKD-3, and 29 participants in ADTKD-4.

### Biomarkers Associated with Worsening CKD Stages

To identify biomarkers associated with CKD progression, we first compared metabolite levels between the control group and ADTKD-1 participants and then between gradually increasing CKD stages (*e.g*., ADTKD-1 versus ADTKD-2, ADTKD-2 versus ADTKD-3, and ADTKD-3 versus ADTKD-4). To evaluate the diagnostic performance of these metabolites to act as early biomarkers and to discriminate the CKD stage, we performed a receiver operating characteristic analysis. Compared with controls, participants in ADTKD-1 stage had increased levels of hexadecanoylglycerol (MG 16:0). ADTKD-2 stage was associated with increased levels of *N*-formylmethionine, *N*-acetylornithine, and pseudouridine and decreased creatine levels. ADTKD-3 stage was associated with a significant increase in levels of 27 metabolites, with pseudouridine showing the best discrimination between groups. ADTKD-4 stage was characterized by a further increase in levels of pseudouridine accompanied by increased levels of *N*6-succinyladenosine, dimethylarginine, and acetylated amino acids. All of these metabolite changes showed good discriminative ability with the area under the receiver operating characteristic curve >0.75 (Figure 3 and Supplemental Figure 3). In total, 30 metabolites showed significant changes in abundance between ADTKD-3 and ADTKD-4 stages, of which 18 were also identified as significantly altered between stages 2 and 3. Enrichment analysis did not identify any metabolite sets that would distinguish the progression from ADTKD-2 stage to ADTKD-3 stage from the later changes (Supplemental Figure 3).

Figure 3**Biomarkers of disease progression.** (A, C, E, G) Volcano plots highlight the differences between patients in stage 1 of the disease and controls (A), stage 2 and stage 1 (C), stage 3 and stage 2 (E), and stage 4 and stage 3 (G). Names in black (in A, C) are shown only in metabolites with nominal *P* value <0.05 and absolute value of log_2_ fold change >0.3. Metabolites in blue and red are significant at FDR-adjusted *P* value <0.05. (B, D, F, H) ROC analysis of the metabolites altered between patients in stage 1 of the disease and controls (B), stage 2 and stage 1 (D), stage 3 and stage 2 (F), and stage 4 and stage 3 (H). ROC curves are shown only for the most significantly changed metabolites at FDR-adjusted *P* value <0.5 (4 metabolites in B), at FDR-adjusted *P* value <0.15 (7 metabolites in D), or the first seven metabolites with the lowest FDR-adjusted *P* value (in F and H). AUC, area under the receiver operating characteristic curve; FPR, false positive rate; TPR, true positive rate.

**Figure:**
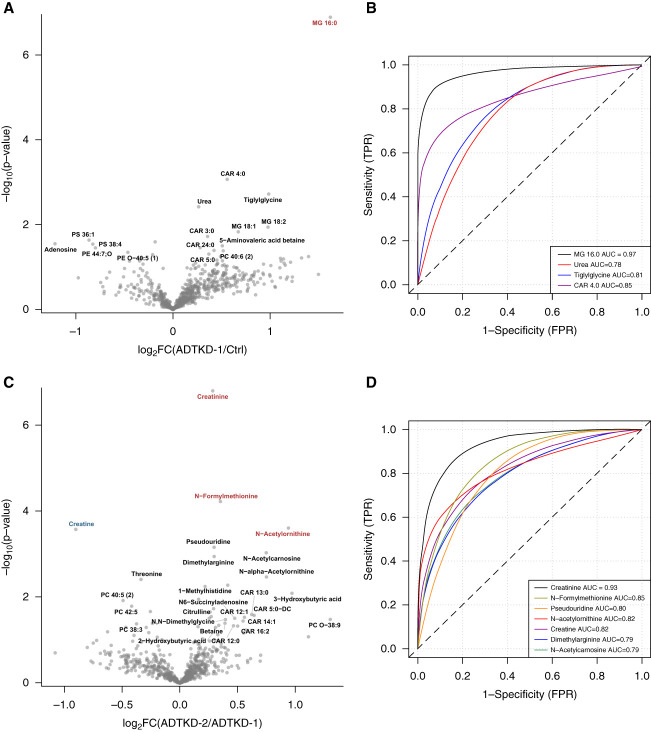

**Figure:**
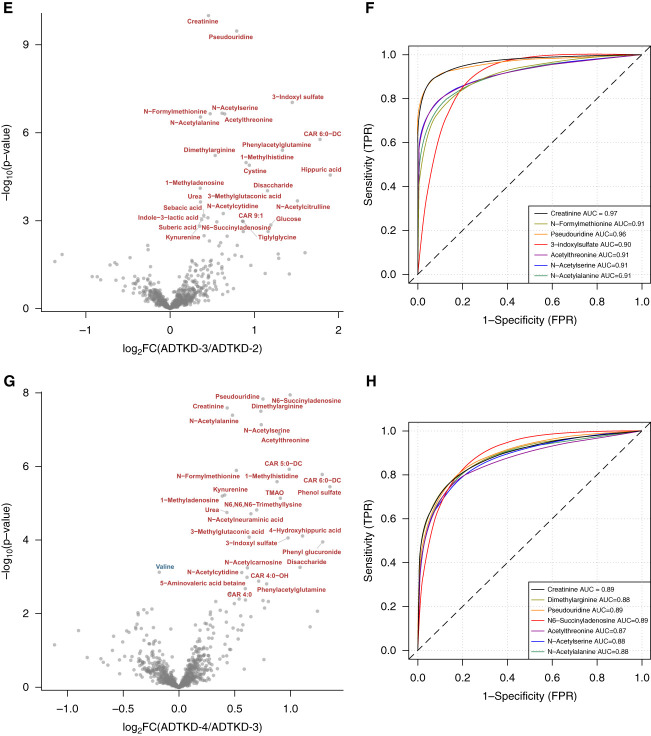

**Figure 4:**
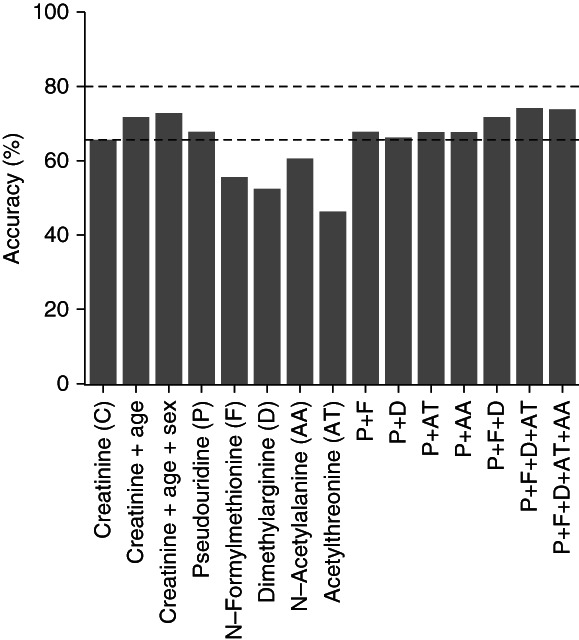
**Accuracy of the individual metabolites and their combinations in the random forest classification of ADTKD stages.** The graph is supplemented with horizontal dashed lines at 65% and 80%.

Finally, we assessed the discriminative power of individual metabolite levels and their combinations for the ADTKD staging using the random forest analysis. This classification method allows for identification of a ranked list of the most important metabolites that can best explain the variation in each outcome even if their relationship is complex and nonlinear.^24^ Consistent with the previous results, pseudouridine, *N*-formylmethionine, acetylthreonine, *N*-acetylalanine, and dimethylarginine were identified as the most informative metabolites for the classification (Supplemental Figure 4, A and B). Random forest analysis showed that pseudouridine alone achieved a classification accuracy of 68%, and combining the five most important metabolites improved the performance to 74% accuracy in ADTKD staging (Figure 4 and Supplemental Table 1). A model using creatinine, age, and sex—the variables included in the CKD-Epidemiology Collaboration equation used for CKD staging—achieved 73% classification accuracy.

Notably, although creatinine levels differed significantly between sexes, the other five key metabolites did not, and their levels were not significantly associated with age (Supplemental Figure 4, C and D).

### Metabolic Profiles, Affected Pathways, and Disease Mechanisms

To identify dysregulated metabolic pathways and mechanisms of ADTKD-*UMOD* and ADTKD-*MUC1* and to assess their relation to kidney disease progression, we specifically compared ADTKD-*UMOD*-4, ADTKD-*MUC1*-4, and the control group that were matched with regard to age and sex composition (with no significant differences between the groups). The *t* test analysis (with false discovery rate-adjusted *P* value <0.05) revealed 84 and 89 metabolites whose levels were significantly different between the controls and ADTKD-*UMOD* stage 4 or ADTKD-*MUC1* stage 4, respectively, with 67 metabolites identified in both comparisons (Figure 2, A and B). The identified changes in metabolite levels correlated strongly between ADTKD-*UMOD* stage 4 and ADTKD-*MUC1* stage 4 groups, further supporting the similarity of their metabolic profiles (Figure 2C, Pearson r=0.89, *P* < 2×10^−16^). Pooling the ADTKD-*UMOD* and ADTKD-*MUC1* stage 4 groups into a single ADTKD-4 group and comparing it with the control group revealed 121 significantly altered metabolites (Supplemental Table 2), whose levels showed significant correlations with eGFR values across all samples, as determined by Spearman correlation analysis (Figure 2D). The distribution of metabolite levels across different ADTKD stage groups, their changes relative to ADTKD-1, and their correlations with eGFR are shown in Supplemental Figures 5–7.

**Figure 5:**
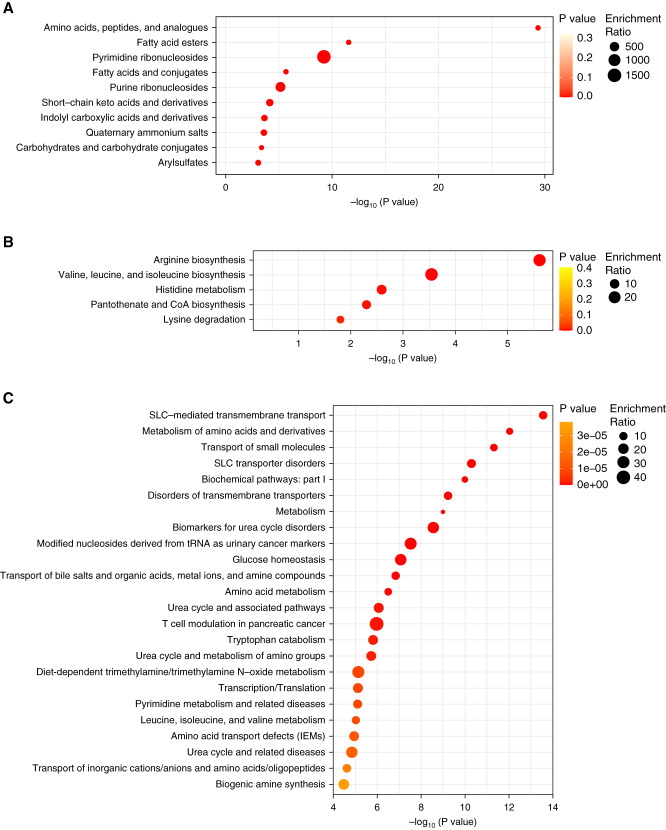
**Enrichment analysis of 121 significant metabolites identified as significantly altered in patients in stage 4 compared with controls using different metabolite set libraries.** (A) Subclasses of chemical structures, FDR-adjusted *P* value <0.1, (B) KEGG database, FDR-adjusted *P* value <0.25, and (C) RaMP-DB, FDR-adjusted *P* value <0.005. Overrepresentation analysis was performed against the reference metabolome on the basis of all of the compounds in the selected library. KEGG, kyoto encyclopedia of genes and genomes; RaMP-DB, Relational database of Metabolomic Pathways; SLC, solute carrier; tRNA, transfer RNA.

**Figure 6:**
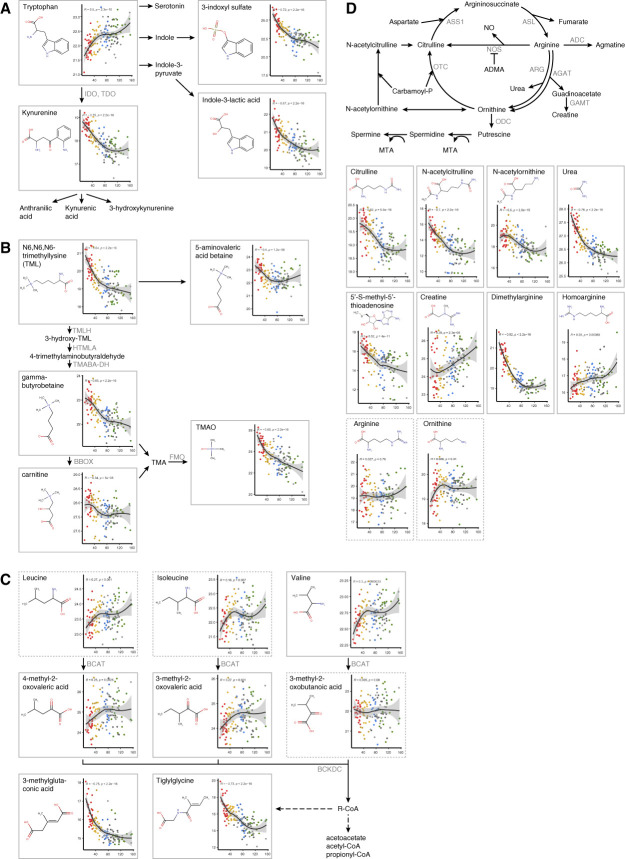
**The metabolites associated with the enriched metabolic pathways and the correlation of their levels (*y*-axis) with eGFR values (*x*-axis).** (A) Pathways involved in tryptophan catabolism. The first and rate-limiting step in the kynurenine pathway is the major catabolic pathway catalyzed by TDO enzyme (liver) or IDO1/IDO2 enzymes (elsewhere). Alternative pathways produce serotonin or 3-indoxyl sulfate and indole-3-lactic acid (*via* the human gut microbial cometabolism). (B) Overview of carnitine synthesis (lysine degradation) and diet-dependent trimethylamine/trimethylamine *N*-oxide metabolism. Trimethylamine produced by bacteria is oxidized to TMAO by FMO enzyme expressed in the liver. (C) Main steps of branched-chain amino acid catabolism. (D) Pathways involved in arginine metabolism. Arginine serves as a precursor for numerous biologically active compounds: nitric oxide, ornithine, agmatine, polyamines, and creatine. Metabolites in dashed rectangles were not significantly altered in stage 4 of ADTKD compared with controls. The color of the nodes corresponds to the sample groups, with red indicating stage 4, yellow stage 3, blue stage 2, green stage 1, and gray the control group. ADC, arginine decarboxylase; ADMA, asymmetric dimethylarginine; AGAT, arginine:glycine amidinotransferase; ARG, arginase; ASL, argininosuccinate lyase; ASS1, argininosuccinate synthase 1; BBOX, *γ*-butyrobetaine dioxygenase; BCAT, branched-chain amino acid transaminase; BCKD, branched-chain keto acid dehydrogenase; FMO, flavin-containing monooxygenase; GAMT, guanidinoacetate *N*-methyltransferase; HTMLA, 3-hydroxy-trimethyllysine aldolase; IDO, indoleamine 2,3-dioxygenase; MTA, 5′-S-methyl-5′-thioadenosine; NO, nitric oxide; NOS, nitric oxide synthase; ODC, ornithine decarboxylase; OTC, ornithine carbamoyltransferase; TDO, tryptophan 2,3-dioxygenase; TMA, trimethylamine; TMABA-DH, 4-trimethylaminobutyraldehyde dehydrogenase; TMAO, trimethylamine *N*-oxide; TMLH, trimethyllysine dioxygenase.

For chemical structures, the metabolomic analysis revealed that worsening CKD stage was significantly associated with altered levels of amino acids, acylcarnitines, fatty acids, pyrimidine and purine ribonucleosides, short-chain keto acids, and phenol and indole derivatives (Figure 5A). Metabolite set enrichment analysis revealed that the most significant alterations were in the biosynthesis of arginine and branched-chain amino acids, carnitine synthesis (lysine degradation), transfer RNA metabolism, tryptophan catabolism, urea cycle, metabolism of amino acids, glucose homeostasis, and solute carrier-mediated transmembrane transport (Figure 5, B and C). The role of corresponding metabolites in the most affected metabolic pathways, along with the correlation of their levels with eGFR, is shown in Figure 6. For most lipids, the metabolite set enrichment analysis on the basis of libraries was not feasible, and they were not included in the enrichment analysis. Several lipid groups had altered levels, mainly oxidized fatty acids, phosphatidylethanolamines, phosphatidylserines, and sphingomyelins.

To gain additional insight into affected metabolic pathways and to identify other functionally related biomarkers, we calculated and compared ratios of all measured polar metabolites (*n*=119). The analysis provided 7021 unique combinations of metabolites that were subsequently correlated with eGFR across all samples (Supplemental Data 3). We searched for metabolite ratios whose correlation with eGFR and discriminative performance were comparable with or better than those of individual metabolites. Through this analysis, we identified the kynurenine/tryptophan and leucine/*γ*-glutamylleucine ratios as providing improved discrimination between individual ADTKD stages (Supplemental Figure 8).

## Discussion

In this retrospective cross-sectional study, we conducted untargeted plasma metabolomics in individuals with ADTKD-*UMOD* or ADTKD-*MUC1*. The primary goal was to identify biomarkers of disease activity to support patient stratification and inform future clinical studies. In addition, we aimed to identify dysregulated metabolic pathways linked to kidney function decline, whether specific to ADTKD subtypes or relevant to CKD broadly. Although ADTKD-*UMOD* and ADTKD-*MUC1* are not fully representative of CKD as a whole, metabolomic analysis of individuals with these genetically defined tubulointerstitial disorders offers a unique opportunity to gain insights into the metabolic correlates of CKD. In contrast to many CKD cohort studies that include patients with heterogeneous and often systemic disease etiologies,^25–27^ our study population is more pathophysiologically uniform, thereby reducing confounding influences on the metabolome. The availability of a genetic diagnosis also enables earlier identification of kidney disease, which is rarely possible in nongenetic forms of CKD. Moreover, because ADTKD-*UMOD* and ADTKD-*MUC1* are tubulointerstitial diseases, the metabolome is not affected by glomerular permeability and less affected by changes in concentration due to variations in serum albumin levels. Because these disorders originate in the kidney, the systemic effects of underlying conditions (*e.g*., diabetes mellitus or lupus) are excluded. However, the plasma levels of metabolites may still be affected by the GFR.

Combining results from four LC-MS platforms, we identified 710 metabolites in plasma samples of 40 genetically unaffected relatives, 51 individuals with ADTKD-*UMOD*, and 49 individuals with ADTKD-*MUC1* at CKD stages 1–4. We compared the metabolite profiles that were obtained using multivariate and univariate analyses. There were no significant differences between the genetically unaffected ADTKD-*UMOD* and ADTKD-*MUC1* relatives and between patients with ADTKD-*UMOD* and ADTKD-*MUC1* at the same stage of the kidney disease. These results suggests that patients with ADTKD-*UMOD* and ADTKD-*MUC1* show more biochemical similarities than differences as they progress across CKD stages, which is consistent with the view that common pathways and pathogenetic processes dominate in the progression of both conditions.

ANOVA analyses identified 95 metabolites whose levels were significantly altered between control and affected samples. The hierarchical clustering analysis suggested that there are no or very few differences in metabolic profiles between controls and patients with ADTKD-*MUC1* and ADTKD-*UMOD* at CKD stages 1 and 2, whereas a number of significant differences exist in and between CKD stages 3 and 4. Using subsequent *t* test–based comparisons between the control group and ADTKD-1 group and then between gradually increasing CKD stages, we identified several metabolites that may act as early biomarkers of disease activity and whose plasma levels discriminate between the stages of the kidney disease.

Plasma from ADTKD-1 participants had increased levels of hexadecanoylglycerol (MG 16:0). There is not much specific information available about hexadecanoylglycerol's role in the kidney; however, increased plasma levels of monoacylglycerols including hexadecanoylglycerol were associated with an increased risk of CKD progression.^28,29^ Plasma metabolites in ADTKD-2 differed from those in ADTKD-1 by increased levels of creatinine, *N*-formylmethionine, *N*-acetylornithine, dimethylarginine, and pseudouridine, and decreased levels of creatine. *N*-formylmethionine is the amino acid active in the initiation of protein synthesis. Increased plasma levels of *N*-formylmethionine indicate mitochondrial dysfunction^30^ and incident renal disease.^31^ *N*-Acetylornithine is an intermediate in the arginine and proline metabolism pathway. Higher circulating levels of several *N*-acetylated amino acids, including *N*-acetylornithine, were associated with kidney failure^32^ and with variants in *NAT8*, a liver- and kidney-specific acetyltransferase gene.^33^ Alternatively, or in parallel, a dysfunction of aminoacylase-1 (ACY1) and aminoacylase-3 (ACY3), which breaks down *N*-acetylated amino acids in the kidney tubular epithelium during intracellular protein catabolism, may also contribute to an increase in acetylated amino acid levels with influence on metabolism and kidney function.^34^ Asymmetric and symmetric dimethylarginines are uremic toxins and mediators of endothelial dysfunction,^35^ and their levels strongly predict CKD progression.^36,37^ Pseudouridine is a modified nucleoside found in various types of RNA. Higher pseudouridine plasma levels are related to transfer RNA catabolism and have been consistently highlighted in metabolomic studies of kidney disease progression.^38^ Levels of these metabolites increased steadily and remained prominent also through the ADTKD-3 and ADTKD-4 stages. In ADTKD-3, there was an increase in acylcarnitines, dicarboxylic fatty acids, and carnitine degradation products, which indicates inefficient fatty acid oxidation, a well-established factor in CKD progression and development of renal fibrosis.^39^ In ADTKD-4, there was a specific increase of succinyladenosine (SAdo) level. SAdo is a dephosphorylated form of succinyladenosine monophosphate and accumulates either with increased flux through the purine nucleotide cycle and fumarate overproduction or in adenylosuccinate lyase deficiency. SAdo has been identified as a new potential biomarker for eGFR estimation and diabetic kidney prognostic assessment.^40^

Searching for metabolite level ratios whose correlation with eGFR and discriminative ability between individual ADTKD stages performed at least as well as those of individual metabolites, we identified the kynurenine-to-tryptophan ratio as a promising biomarker discriminating stage 1 from stages 2 and 3. The kynurenine-to-tryptophan ratio reflects the activity of indoleamine 2,3-dioxygenase, an inflammation-induced and rate-limiting enzyme of tryptophan metabolism that catalyzes the degradation of tryptophan to kynurenine.^41^ Increases in the kynurenine-to-tryptophan ratio thus may inform us about the start and intensity of the inflammatory processes in the kidney.^42^ Several studies have implicated dysregulation of the tryptophan–kynurenine pathway in CKD.^41,43^ Notably, an 8 year follow-up study of 1741 patients from the Korean Genome Epidemiology Study identified the kynurenine-to-tryptophan ratio as a predictor of future CKD development. In addition, modulation of kynurenine levels has been observed after renin–angiotensin system inhibition in CKD populations, both with and without type 2 diabetes mellitus.^44^ The association of the kynurenine-to-tryptophan ratio with CKD progression in individuals whose disease is directly linked to specific genetic defects and confined to the kidney suggests that this association is independent of nonrenal effects from systemic conditions such as diabetic nephropathy or cardiovascular disease. Furthermore, the discovery of this ratio through an unbiased, untargeted metabolomic approach strengthens the validity and potential mechanistic relevance of this finding.

With CKD progression, the metabolome undergoes a range of dynamic changes. To better understand this process, we mapped the most significantly altered metabolites to known metabolic pathways. Our cohort consisted of two genetically stratified groups of patients with kidney disease caused by intracellular deposition of the mutant proteins, leading to progressive tubular cell injury. Early in the disease course, we observed alterations in amino acid metabolism, signs of mitochondrial dysfunction affecting energy metabolism, and accelerated nucleic acid catabolism. These changes were accompanied by metabolic indicators of inflammation. Importantly, the affected metabolites and pathways were not specific to ADTKD-*UMOD* or ADTKD-*MUC1* but rather reflected common metabolic signatures of CKD that have also been reported in other studies, regardless of disease etiology.^45^

Although we also aimed to identify metabolic profiles capable of distinguishing ADTKD-*UMOD* from ADTKD-*MUC1* and other forms of CKD, we did not observe clear or consistent differences to support such separation. Instead, the metabolic alterations observed in ADTKD are largely consistent with those reported in metabolomic studies of CKD more broadly.^45^ Thus, although ADTKD is a tubulointerstitial kidney disease rather than glomerular, the effects on the metabolomic pathways appear similar. Although the identification of individual metabolites as biomarkers of CKD in ADTKD would be especially beneficial, we can also study the effect of diets^46,47^ and metabolic interventions on the metabolome of ADTKD as a whole in future work and see the holistic effect of different therapeutic interventions.

In summary, in this discovery study, we identified several promising metabolite biomarkers of ADTKD-*UMOD* and ADTKD-*MUC1* disease activity. Some of these biomarkers, such as hexadecanoylglycerol (MG 16:0), may potentially identify kidney disease activity early before eGFR decline. Some of them, such as *N*-formylmethionine, dimethylarginine, and pseudouridine, highly correlate with eGFR. Others, such as an increased kynurenine-to-tryptophan ratio and increased acylcarnitines levels, are specifically informative of progression from stage 2 to 3 and increased SAdo levels on progression to stage 4.

A key limitation of this study is that kidney disease status was assessed solely on the basis of creatinine measurements and creatinine-derived CKD staging. Although this approach reflects current clinical standards, it inherently limits our ability to identify biomarkers that definitively outperform creatinine or eGFR in monitoring disease progression in ADTKD. Consequently, our study represents an initial exploratory step to identify candidate metabolic biomarkers associated with CKD stages, rather than providing conclusive evidence of superiority over existing markers. In addition, no adjustments were made for potential confounding factors such as diet or medication use, which may influence metabolite levels. The clinical utility and validity of these biomarker signatures for patient stratification, treatment selection, and outcome prediction require additional investigation. Future retrospective and prospective studies using targeted metabolite assays in larger, independent ADTKD-*UMOD* and ADTKD-*MUC1* cohorts will be essential to validate and extend these findings, ideally incorporating longitudinal clinical outcomes beyond creatinine-based assessments.

## Supplementary Material

## Data Availability

All original data, including deidentified patient-level data or individual laboratory data measurements, are included in the manuscript and/or supplemental material.

## Declarative Statements

This study includes clinical experimentation and received Institutional Review Board or Ethics Committee approval. All patients provided written informed consent. This study includes clinical experimentation and complies with the Declaration of Helsinki.

## Supplemental Material

Supplemental Figure 1. Multivariate analyses (PCA and OPLS).

Supplemental Figure 2. ANOVA analysis.

Supplemental Figure 3. Receiver operating characteristic curve values and enrichment analysis of the metabolites that discriminate best between the stages of the disease.

Supplemental Figure 4. Random forest analyses.

Supplemental Figure 5. Values measured in 121 metabolites identified as significantly altered in patients in stage 4 compared with controls.

Supplemental Figure 6. Log_2_FC values measured in 121 metabolites identified as significantly altered in patients in stage 4 compared with controls.

Supplemental Figure 7. Values measured in 121 metabolites (*y*-axis, arbitrary units) identified as significantly altered in patients in stage 4 compared with controls are correlated with eGFR values (on *x*-axis).

Supplemental Figure 8. Performance of selected metabolite ratios.

Supplemental Table 1. Performance of the individual metabolites and their combinations in the random forest classification of ADTKD stages.

Supplemental Table 2. The list of 121 significantly changed metabolites in the comparison of ADTKD stage 4 versus controls.

Supplemental Data 1. Results of LC-MS analysis.

Supplemental Data 2. Results of ANOVA analysis and Tukey HSD *post-hoc* tests in 95 metabolites with significantly altered levels.

Supplemental Data 3. Correlation of metabolite ratios with eGFR.

## References

[1] Živná M Kidd KO Barešová V Hůlková H Kmoch S Bleyer AJ Sr. Autosomal dominant tubulointerstitial kidney disease: a review. Am J Med Genet C Semin Med Genet. 2022;190(3):309–324. doi:10.1002/ajmg.c.32008 36250282 PMC9619361

[2] Olinger E Hofmann P Kidd K, . Clinical and genetic spectra of autosomal dominant tubulointerstitial kidney disease due to mutations in UMOD and MUC1. Kidney Int. 2020;98(3):717–731. doi:10.1016/j.kint.2020.04.038 32450155

[3] Hart TC Gorry MC Hart PS, . Mutations of the UMOD gene are responsible for medullary cystic kidney disease 2 and familial juvenile hyperuricaemic nephropathy. J Med Genet. 2002;39(12):882–892. doi:10.1136/jmg.39.12.882 12471200 PMC1757206

[4] Kirby A Gnirke A Jaffe DB, . Mutations causing medullary cystic kidney disease type 1 lie in a large VNTR in MUC1 missed by massively parallel sequencing. Nat Genet. 2013;45(3):299–303. doi:10.1038/ng.2543 23396133 PMC3901305

[5] Nanamatsu A de Araújo L LaFavers KA El-Achkar TM. Advances in uromodulin biology and potential clinical applications. Nat Rev Nephrol. 2024;20(12):806–821. doi:10.1038/s41581-024-00881-7 39160319 PMC11568936

[6] Al-Bataineh MM Sutton TA Hughey RP. Novel roles for mucin 1 in the kidney. Curr Opin Nephrol Hypertens. 2017;26(5):384–391. doi:10.1097/MNH.0000000000000350 28622163 PMC5786376

[7] Eckardt KU Alper SL Antignac C, . Autosomal dominant tubulointerstitial kidney disease: diagnosis, classification, and management-A KDIGO consensus report. Kidney Int. 2015;88(4):676–683. doi:10.1038/ki.2015.28 25738250

[8] Dvela-Levitt M Kost-Alimova M Emani M, . Small molecule targets TMED9 and promotes lysosomal degradation to reverse proteinopathy. Cell. 2019;178(3):521–535 e23. doi:10.1016/j.cell.2019.07.002 31348885

[9] Trudu M Schaeffer C Riba M, . Early involvement of cellular stress and inflammatory signals in the pathogenesis of tubulointerstitial kidney disease due to UMOD mutations. Sci Rep. 2017;7(1):7383. doi:10.1038/s41598-017-07804-6 28785050 PMC5547146

[10] Bazua-Valenti S Brown MR Zavras J, . Disrupted uromodulin trafficking is rescued by targeting TMED cargo receptors. J Clin Invest. 2024;134(24):e180347. doi:10.1172/JCI180347 39680459 PMC11645142

[11] Kim Y Li C Gu C, . MANF stimulates autophagy and restores mitochondrial homeostasis to treat autosomal dominant tubulointerstitial kidney disease in mice. Nat Commun. 2023;14(1):6493. doi:10.1038/s41467-023-42154-0 37838725 PMC10576802

[12] Dvela-Levitt M Shaw JL Greka A. A rare kidney disease to cure them all? towards mechanism-based therapies for proteinopathies. Trends Mol Med. 2021;27(4):394–409. doi:10.1016/j.molmed.2020.11.008 33341352

[13] Peek JL Wilson MH. Cell and gene therapy for kidney disease. Nat Rev Nephrol. 2023;19(7):451–462. doi:10.1038/s41581-023-00702-3 36973494 PMC10339687

[14] Tekendo-Ngongang C Gleeson JG Mignon L. Treating the untreatable: antisense oligonucleotides as an individualized therapy for rare genetic kidney diseases. J Am Soc Nephrol. 2024;35(12):1774–1777. doi:10.1681/ASN.0000000532 39331470 PMC11617478

[15] Kidd K Vylet'al P Schaeffer C, . Genetic and clinical predictors of age of ESKD in individuals with autosomal dominant tubulointerstitial kidney disease due to UMOD mutations. Kidney Int Rep. 2020;5(9):1472–1485. doi:10.1016/j.ekir.2020.06.029 32954071 PMC7486199

[16] Bleyer AJ Kmoch S Antignac C, . Variable clinical presentation of an MUC1 mutation causing medullary cystic kidney disease type 1. Clin J Am Soc Nephrol. 2014;9(3):527–535. doi:10.2215/CJN.06380613 24509297 PMC3944763

[17] Bleyer AJ Kidd K Robins V, . Outcomes of patient self-referral for the diagnosis of several rare inherited kidney diseases. Genet Med. 2020;22(1):142–149. doi:10.1038/s41436-019-0617-8 31337885 PMC6946861

[18] Vylet'al P Kublová M Kalbácová M, . Alterations of uromodulin biology: a common denominator of the genetically heterogeneous FJHN/MCKD syndrome. Kidney Int. 2006;70(6):1155–1169. doi:10.1038/sj.ki.5001728 16883323

[19] Živná M Kidd K Přistoupilová A, . Noninvasive immunohistochemical diagnosis and novel MUC1 mutations causing autosomal dominant tubulointerstitial kidney disease. J Am Soc Nephrol. 2018;29(9):2418–2431. doi:10.1681/ASN.2018020180 29967284 PMC6115665

[20] Blumenstiel B DeFelice M Birsoy O, . Development and validation of a mass spectrometry-based assay for the molecular diagnosis of mucin-1 kidney disease. J Mol Diagn. 2016;18(4):566–571. doi:10.1016/j.jmoldx.2016.03.003 27157321

[21] Hricko J Rudl Kulhava L Paucova M, . Short-term stability of serum and liver extracts for untargeted metabolomics and lipidomics. Antioxidants (Basel). 2023;12(5):986. doi:10.3390/antiox12050986 37237852 PMC10215277

[22] Tsugawa H Ikeda K Takahashi M, . A lipidome atlas in MS-DIAL 4. Nat Biotechnol. 2020;38(10):1159–1163. doi:10.1038/s41587-020-0531-2 32541957

[23] Pang Z Lu Y Zhou G, . MetaboAnalyst 6.0: towards a unified platform for metabolomics data processing, analysis and interpretation. Nucleic Acids Res. 2024;52(W1):W398–W406. doi:10.1093/nar/gkae253 38587201 PMC11223798

[24] Antonelli J Claggett BL Henglin M, . Statistical workflow for feature selection in human metabolomics data. Metabolites 2019;9(7):143. doi:10.3390/metabo9070143 31336989 PMC6680705

[25] Steinbrenner I Schultheiss UT Bächle H, . Associations of urine and plasma metabolites with kidney failure and death in a chronic kidney disease cohort. Am J Kidney Dis. 2024;84(4):469–481. doi:10.1053/j.ajkd.2024.03.028 38815646

[26] Shah VO Townsend RR Feldman HI Pappan KL Kensicki E Vander Jagt DL. Plasma metabolomic profiles in different stages of CKD. Clin J Am Soc Nephrol. 2013;8(3):363–370. doi:10.2215/CJN.05540512. 23220422 PMC3586968

[27] Rhee EP. Metabolomics and renal disease. Curr Opin Nephrol Hypertens. 2015;24(4):371–379. doi:10.1097/MNH.0000000000000136 26050125 PMC4479960

[28] Afshinnia F Rajendiran TM Wernisch S, . Lipidomics and biomarker discovery in kidney disease. Semin Nephrol. 2018;38(2):127–141. doi:10.1016/j.semnephrol.2018.01.004 29602396 PMC5881936

[29] Afshinnia F Rajendiran TM Karnovsky A, . Lipidomic signature of progression of chronic kidney disease in the chronic renal insufficiency cohort. Kidney Int Rep. 2016;1(4):256–268. doi:10.1016/j.ekir.2016.08.007 28451650 PMC5402253

[30] Rogers RS Sharma R Shah HB, . Circulating N-lactoyl-amino acids and N-formyl-methionine reflect mitochondrial dysfunction and predict mortality in septic shock. Metabolomics. 2024;20(2):36. doi:10.1007/s11306-024-02089-z 38446263 PMC10917846

[31] Cai N Gomez-Duran A Yonova-Doing E, . Mitochondrial DNA variants modulate N-formylmethionine, proteostasis and risk of late-onset human diseases. Nat Med. 2021;27(9):1564–1575. doi:10.1038/s41591-021-01441-3 34426706

[32] Suhre K Shin SY Petersen AK, . Human metabolic individuality in biomedical and pharmaceutical research. Nature. 2011;477(7362):54–60. doi:10.1038/nature10354 21886157 PMC3832838

[33] Luo S Surapaneni A Zheng Z, . NAT8 variants, N-acetylated amino acids, and progression of CKD. Clin J Am Soc Nephrol. 2020;16(1):37–47. doi:10.2215/CJN.08600520 33380473 PMC7792648

[34] Luo S Feofanova EV Tin A, . Genome-wide association study of serum metabolites in the African American study of kidney disease and hypertension. Kidney Int. 2021;100(2):430–439. doi:10.1016/j.kint.2021.03.026 33838163 PMC8583323

[35] Oliva-Damaso E Oliva-Damaso N Rodriguez-Esparragon F, . Asymmetric (ADMA) and symmetric (SDMA) dimethylarginines in chronic kidney disease: a clinical approach. Int J Mol Sci. 2019;20(15):3668. doi:10.3390/ijms20153668 31357472 PMC6696355

[36] Caglar K Yilmaz MI Sonmez A, . ADMA, proteinuria, and insulin resistance in non-diabetic stage I chronic kidney disease. Kidney Int. 2006;70(4):781–787. doi:10.1038/sj.ki.5001632 16820789

[37] Au AYM Mantik K Bahadory F, . Plasma arginine metabolites in health and chronic kidney disease. Nephrol Dial Transplant. 2023;38(12):2767–2775. doi:10.1093/ndt/gfad108 37230955

[38] Wen DH Zheng ZH Surapaneni A, . Metabolite profiling of CKD progression in the chronic renal insufficiency cohort study. JCI Insight. 2022;7(20):e161696. doi:10.1172/jci.insight.161696 36048534 PMC9714776

[39] Allison SJ. Fibrosis: dysfunctional fatty acid oxidation in renal fibrosis. Nat Rev Nephrol. 2015;11(2):64. doi:10.1038/nrneph.2014.244 25536395

[40] Jiang JJ Sham TT Gu XF, . Insights into serum metabolic biomarkers for early detection of incident diabetic kidney disease in Chinese patients with type 2 diabetes by random forest. Aging (Albany NY). 2024;16(4):3420–3530. doi:10.18632/aging.205542 38349886 PMC10929832

[41] Mor A Kalaska B Pawlak D. Kynurenine pathway in chronic kidney disease: what's old, what's new, and what's next? Int J Tryptophan Res. 2020;13:1178646920954882. doi:10.1177/1178646920954882 35210786 PMC8862190

[42] Wee HN Liu JJ Ching J Kovalik JP Lim SC. The kynurenine pathway in acute kidney injury and chronic kidney disease. Am J Nephrol. 2021;52(10-11):771–787. doi:10.1159/000519811 34753140 PMC8743908

[43] Dahabiyeh LA Nimer RM Sumaily KM, . Metabolomics profiling distinctively identified end-stage renal disease patients from chronic kidney disease patients. Sci Rep. 2023;13(1):6161. doi:10.1038/s41598-023-33377-8 37061630 PMC10105740

[44] Cernaro V Loddo S Macaione V, . RAS inhibition modulates kynurenine levels in a CKD population with and without type 2 diabetes mellitus. Int Urol Nephrol. 2020;52(6):1125–1133. doi:10.1007/s11255-020-02469-z 32314169

[45] Moritz L Schumann A Pohl M Köttgen A Hannibal L Spiekerkoetter U. A systematic review of metabolomic findings in adult and pediatric renal disease. Clin Biochem. 2024;123:110703. doi:10.1016/j.clinbiochem.2023.110703 38097032

[46] Rovira J Ramirez-Bajo MJ Banon-Maneus E, . Mediterranean diet pattern: potential impact on the different altered pathways related to cardiovascular risk in advanced chronic kidney disease. Nutrients. 2024;16(21):3739. doi:10.3390/nu16213739 39519573 PMC11547550

[47] Cabała S Herosimczyk A. Diet-induced proteomic and metabolomic signatures in chronic kidney disease: a precision nutrition approach. Metabolites. 2025;15(3):211. doi:10.3390/metabo15030211 40137175 PMC11943711

