# Supplementary material for: Plasma Metabolites Associated with CKD Stage in Autosomal Dominant Tubulointerstitial Kidney Disease
Source: Kidney360. 2025 Nov 14;7(2):321–34. doi: 10.34067/KID.0000001029 (PMC12935368; doi:10.34067/KID.0000001029)
Supplement: Supplementary file 5 [file kidney360-7-321-s005.pdf]

Supplemental Material

This appendix has been provided by the authors to give readers additional information about their work.

Supplement to: Musalkova D. et al.: Plasma Metabolites Associated with CKD Stage in ADTKD

Table of contents:

Supplemental Figure 1: Multivariate analyses (PCA and OPLS). *(p. 2)*

Supplemental Figure 2: ANOVA analysis. *(p. 3)*

Supplemental Figure 3: AUC values and enrichment analysis of the metabolites that discriminate best between the stages of the disease. *(p. 4)*

Supplemental Figure 4: Random forest analyses. *(p. 5)*

Supplemental Figure 5: Values measured in 121 metabolites identified as significantly altered in stage 4 patients compared to controls. *(p. 6)*

Supplemental Figure 6: Log_2FC values measured in 121 metabolites identified as significantly altered in stage 4 patients compared to controls. *(p. 7)*

Supplemental Figure 7: Values measured in 121 metabolites (y-axis, arbitrary units) identified as significantly altered in stage 4 patients compared to controls are correlated with eGFR values (on x-axis). *(p. 8)*

Supplemental Figure 8: Performance of selected metabolite ratios. *(p. 9)*

Supplemental Table 1: Performance of the individual metabolites and their combinations in the random forest classification of ADTKD stages. *(p. 10)*

Supplemental Table 2: The list of 121 significantly changed metabolites in comparison of ADTKD stage 4 vs. controls. *(p. 11-12)*

Supplemental Methods *(p.13-14)*

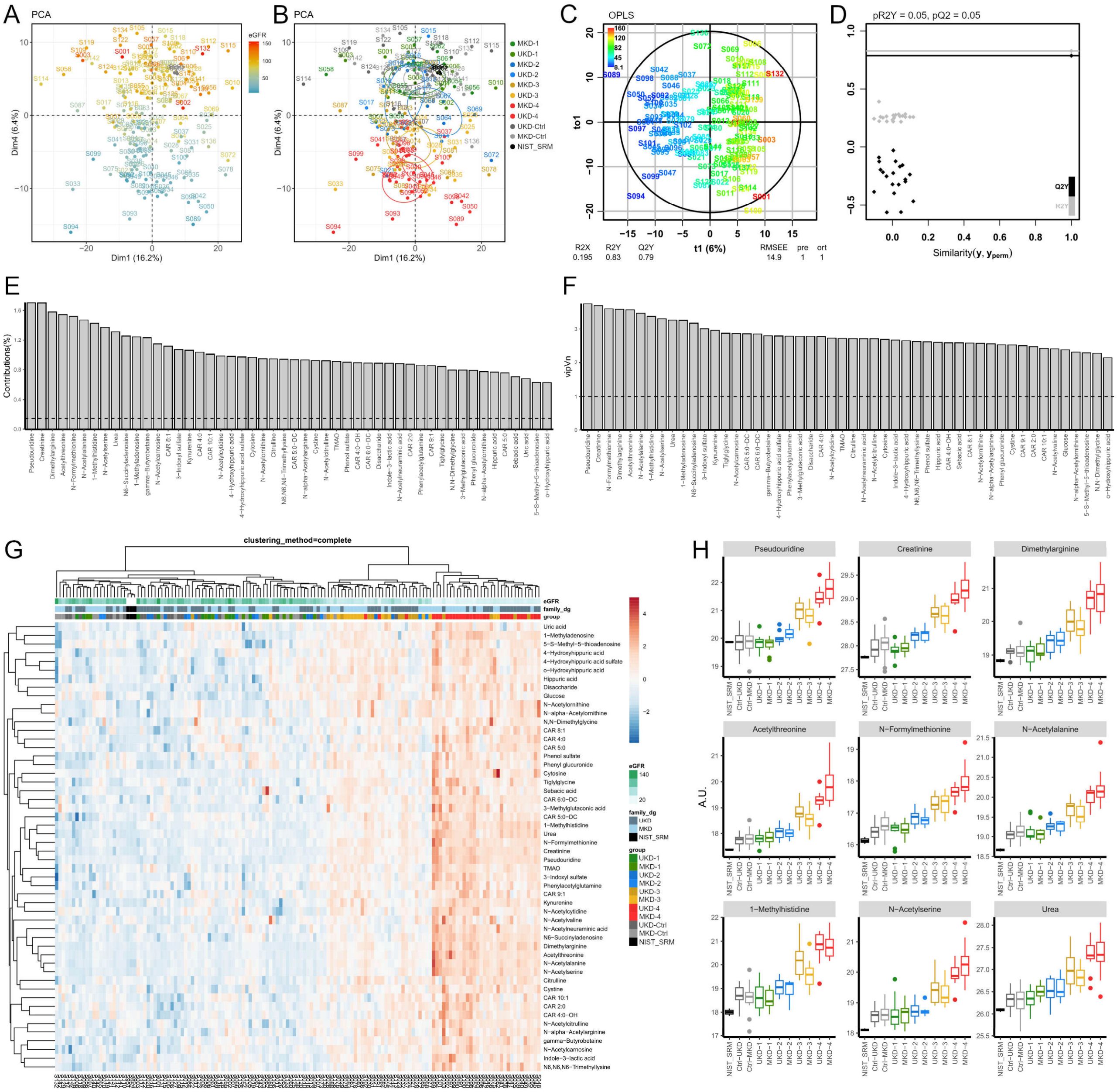

Supplemental Figure 1: Multivariate analyses (PCA and OPLS). (A) PCA analysis shows the separation of samples across the principal component 4 (6.4% of total variance) as a function of eGFR values. Controls are clearly separated from the patients in stage 4 of the kidney disease (B). (C) OPLS analysis shows that the metabolic phenotypes of the samples differ depending on the eGFR values. Corresponding values of R2X, R2Y and model predictive ability (Q2Y) are shown below the plot. (D) Significance diagnostics; the R2Y and Q2Y of the model are compared with the corresponding values obtained after random permutation of the y response. (E) Top 50 contributors to the separation of samples across PC4 from PCA analysis. (F) Top 50 contributors with the highest VIP values from the OPLS model. (G) Hierarchical clustering of samples based on the values of the top 50 contributors identified by PCA and OPLS analysis. (H) Values measured in the top 9 contributors identified by PCA and OPLS analysis (values are expressed as arbitrary units, A.U.). MKD, ADTKD-*MUC1*; UKD, ADTKD-*UMOD*; Ctrl-UKD, controls coming from ADTKD-*UMOD* families; Ctrl-MKD, controls coming from ADTKD-*MUC1* families; NIST_SRM, NIST standard reference material.

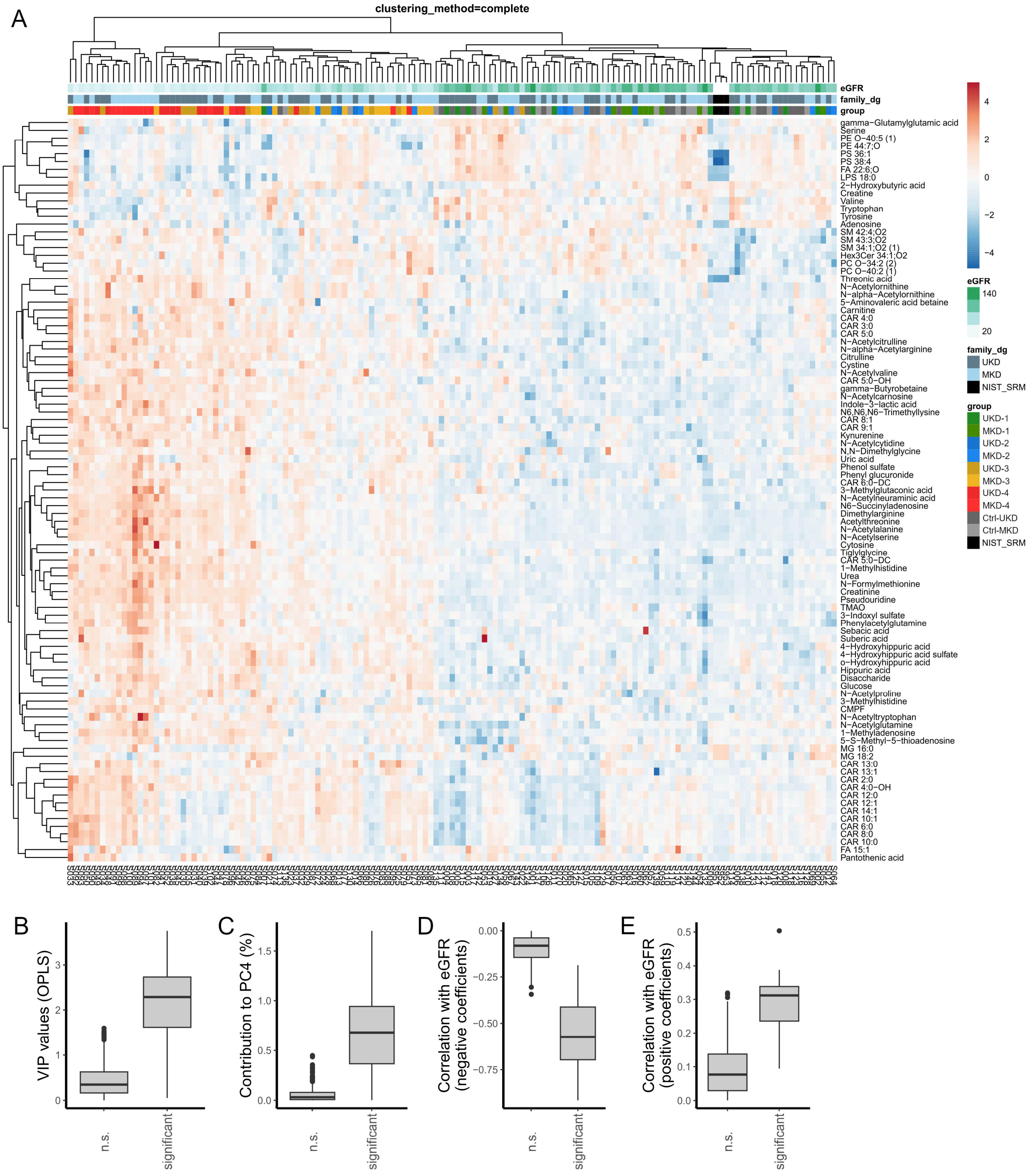

Supplemental Figure 2: ANOVA analysis. (A) Hierarchical clustering of samples based on the metabolite values identified as significant by ANOVA analysis (FDR-adjusted P-value <0.05). (B) Distribution of VIP values from OPLS analysis in metabolites found significant by ANOVA analysis and not significant metabolites (n.s.). (C) Distribution of the contributions to principal component 4 in PCA analysis in metabolites found significant by ANOVA analysis and not significant metabolites (n.s.). (D,E) Distribution of the Spearman correlation coefficients (from the correlation with eGFR) in metabolites found significant by ANOVA analysis and not significant metabolites (n.s.). Metabolites with negative Spearman correlation coefficients are shown in (D), and metabolites with positive Spearman correlation coefficients are shown in (E). MKD, ADTKD-*MUC1*; UKD, ADTKD-*UMOD*; Ctrl-UKD, controls coming from ADTKD-*UMOD* families; Ctrl-MKD, controls coming from ADTKD-*MUC1* families; NIST_SRM, NIST SRM 1950 standard reference material; VIP, Variable Importance in Projection.

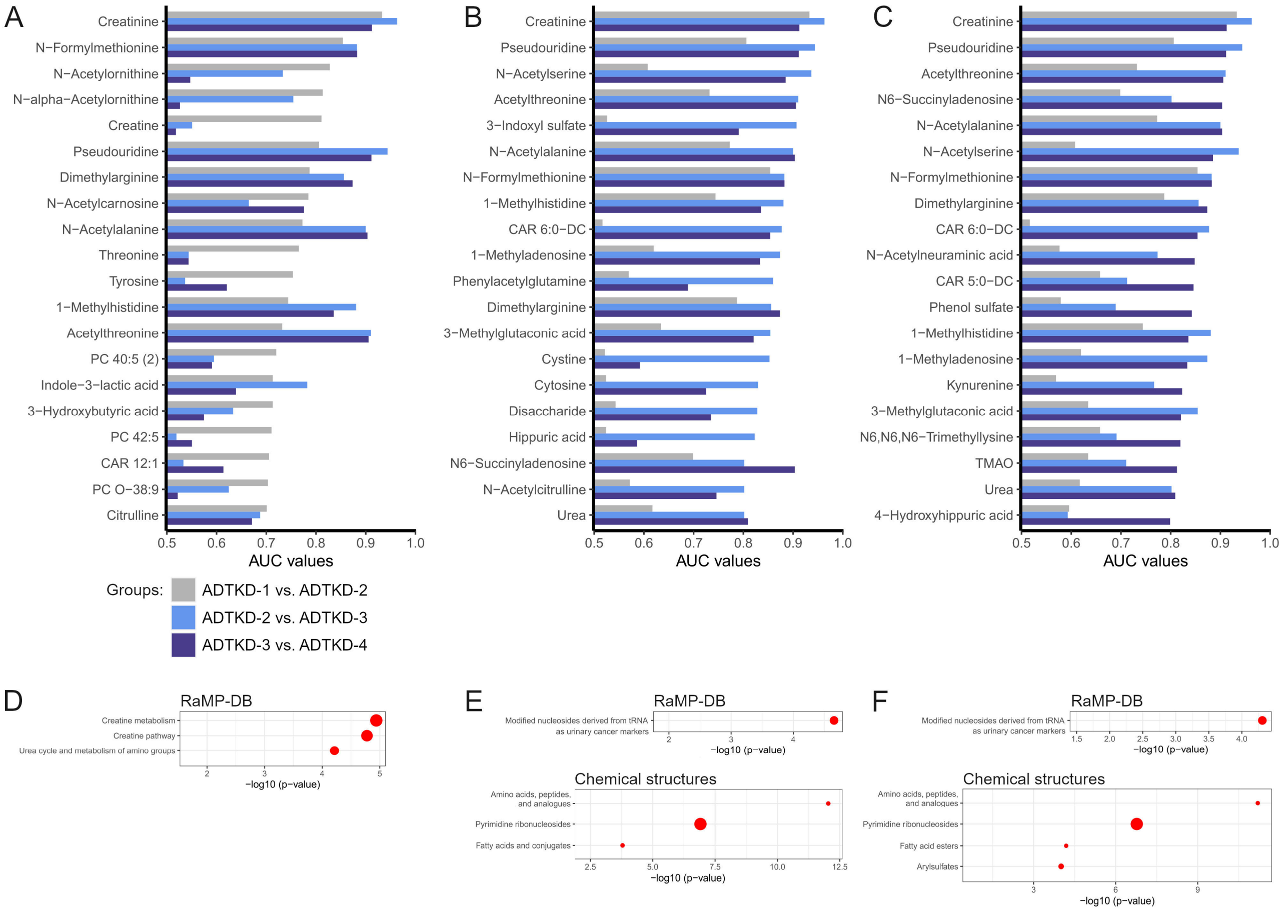

Supplemental Figure 3: AUC values and enrichment analysis of the metabolites that discriminate best between the stages of the disease. (A,B,C) AUC values from ROC analysis of the metabolites that differ between the groups of patients in different stages. Bar plots show the first 20 metabolites with the best AUC values when comparing stage 1 vs. stage 2 (A), stage 2 vs. stage 3 (B), and stage 3 vs. stage 4 (C). (D,E,F) Enrichment analysis of the 4, 27, and 30 metabolites that were identified as singificantly changed when comparing stage 1 vs. stage 2 (D), stage 2 vs. stage 3 (E), and stage 3 vs. stage 4 (F), respectively. Enrichment was performed using different metabolite set libraries; RaMP-DB, FDR adjusted *P*-value < 0.2; sub-classes of chemical structures, FDR adjusted *P*-value < 0.05; KEGG database, FDR adjusted *P*-value < 0.2. Overrepresentation analysis was performed against reference metabolome based on all the compounds in the selected library. The size of the dots is proportional to the enrichment ratio of the metabolite set. The enrichment ratio is calculated as the number of hits within a particular metabolic pathway divided by the expected number of hits.

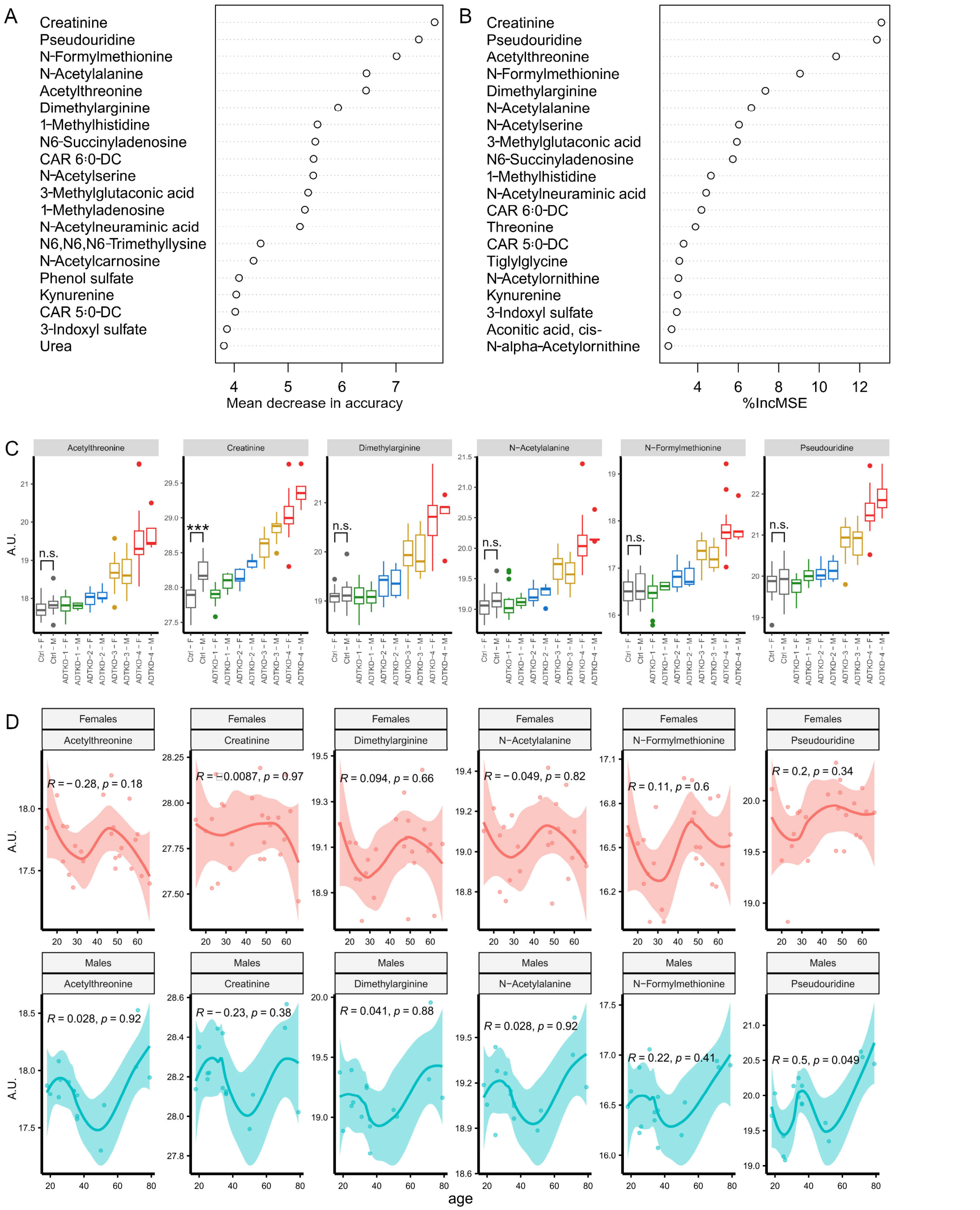

Supplemental Figure 4: Random forest analyses. Random forest analyses showing the top 20 metabolites ordered by the importance for separation between ADTKD stages (A) or for separation according to the eGFR values (B). (C) The distribution of measured values in male and female controls or patients in the corresponding stages. The values in female controls (Ctrl – F) and male controls (Ctrl – M) were using the Student´s t-test. (D) The values measured in female and male controls (y-axis, arbitrary units) were correlated with the age of the controls (on x-axis). The color of the dots corresponds to the sex. The graphs are supplemented with loess curves and Spearman correlation coefficients with corresponding *P*-values (at the top of each panel). ***, *P* << 0.001; n.s., not significant; %IncMSE, percentage of the increase in mean squared error; F, female; M, male.

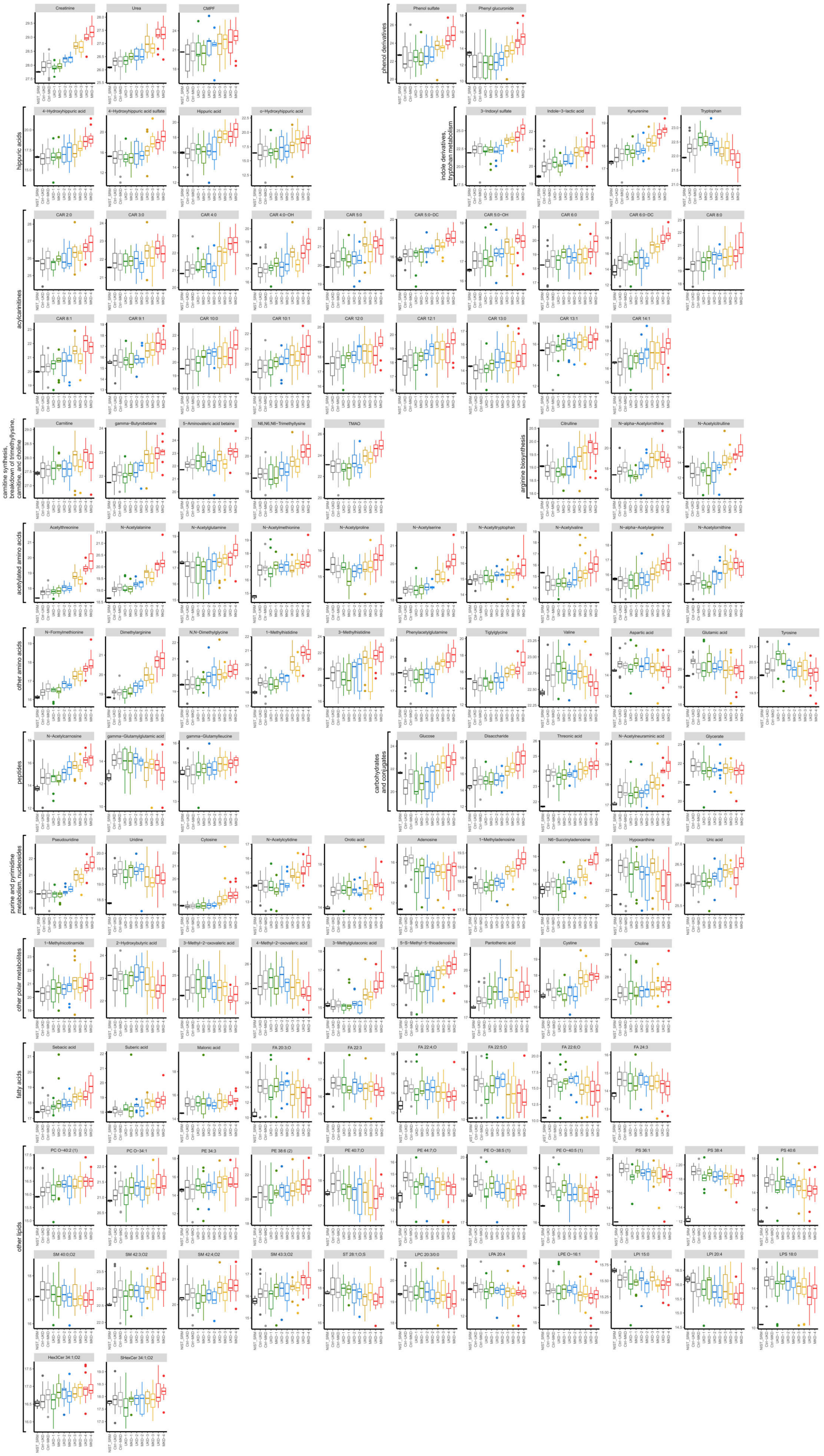

Supplemental Figure 5: Values measured in 121 metabolites identified as significantly altered in stage 4 patients compared to controls. The values are expressed in arbitrary units (y-axis) and displayed as boxplots. Samples are grouped according to the stages of the kidney disease and the genotypes. MKD, ADTKD-*MUC1*; UKD, ADTKD-*UMOD*; Ctrl-UKD, controls coming from ADTKD-*UMOD* families; Ctrl-MKD, controls coming from ADTKD-*MUC1* families; NIST_SRM, NIST SRM 1950 standard reference material.

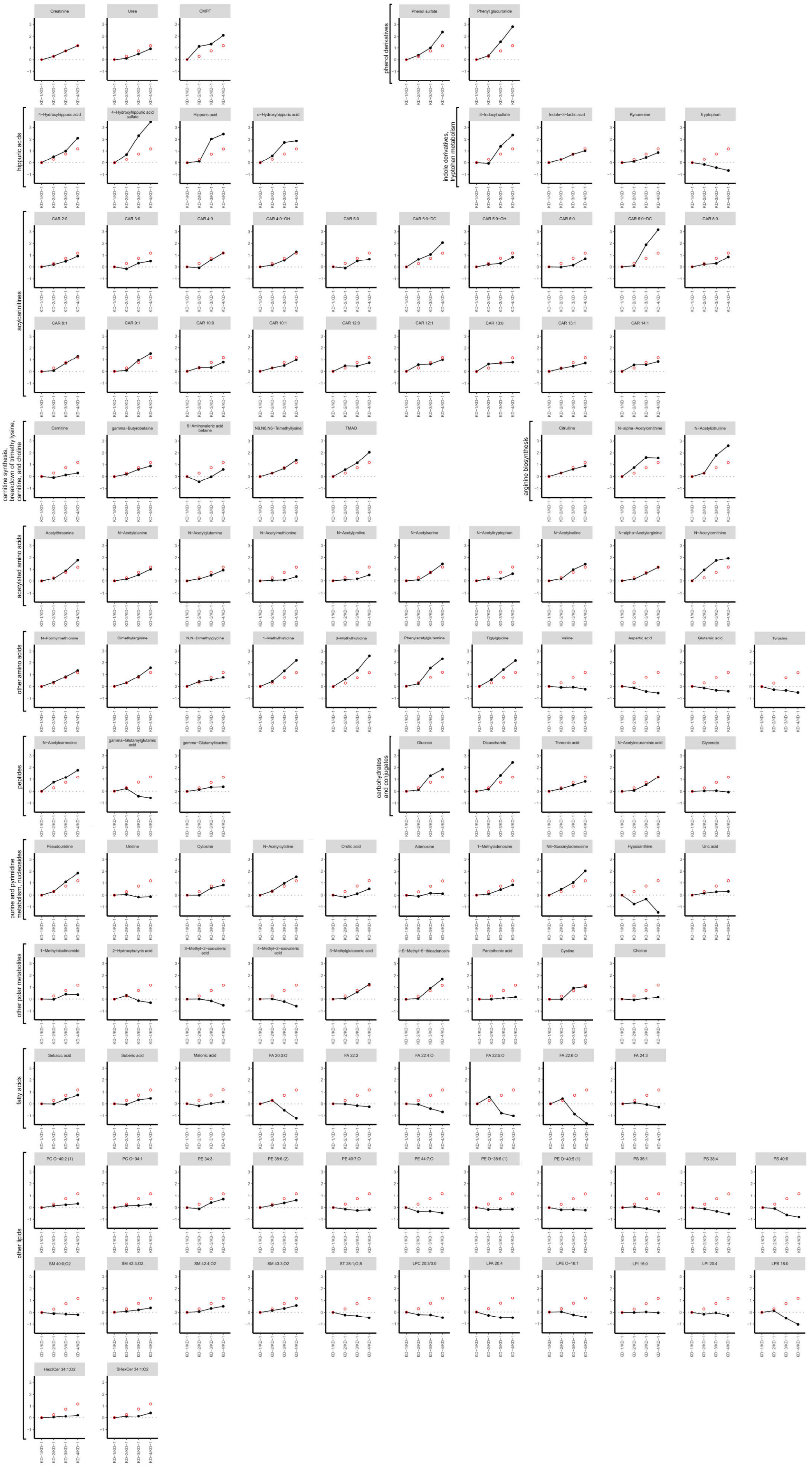

Supplemental Figure 6: Log$_2$FC values measured in 121 metabolites identified as significantly altered in stage 4 patients compared to controls. The results are expressed as the average value in the respective group of patients (ADTKD-1, ADTKD-2, ADTKD-3, or ADTKD-4) relative to the group ADTKD-1. The black color corresponds to the metabolite in the box, and the red color corresponds to the creatinine. KD-1, ADTKD-1; KD-2, ADTKD-2; KD-3, ADTKD-3; KD-4, ADTKD-4.

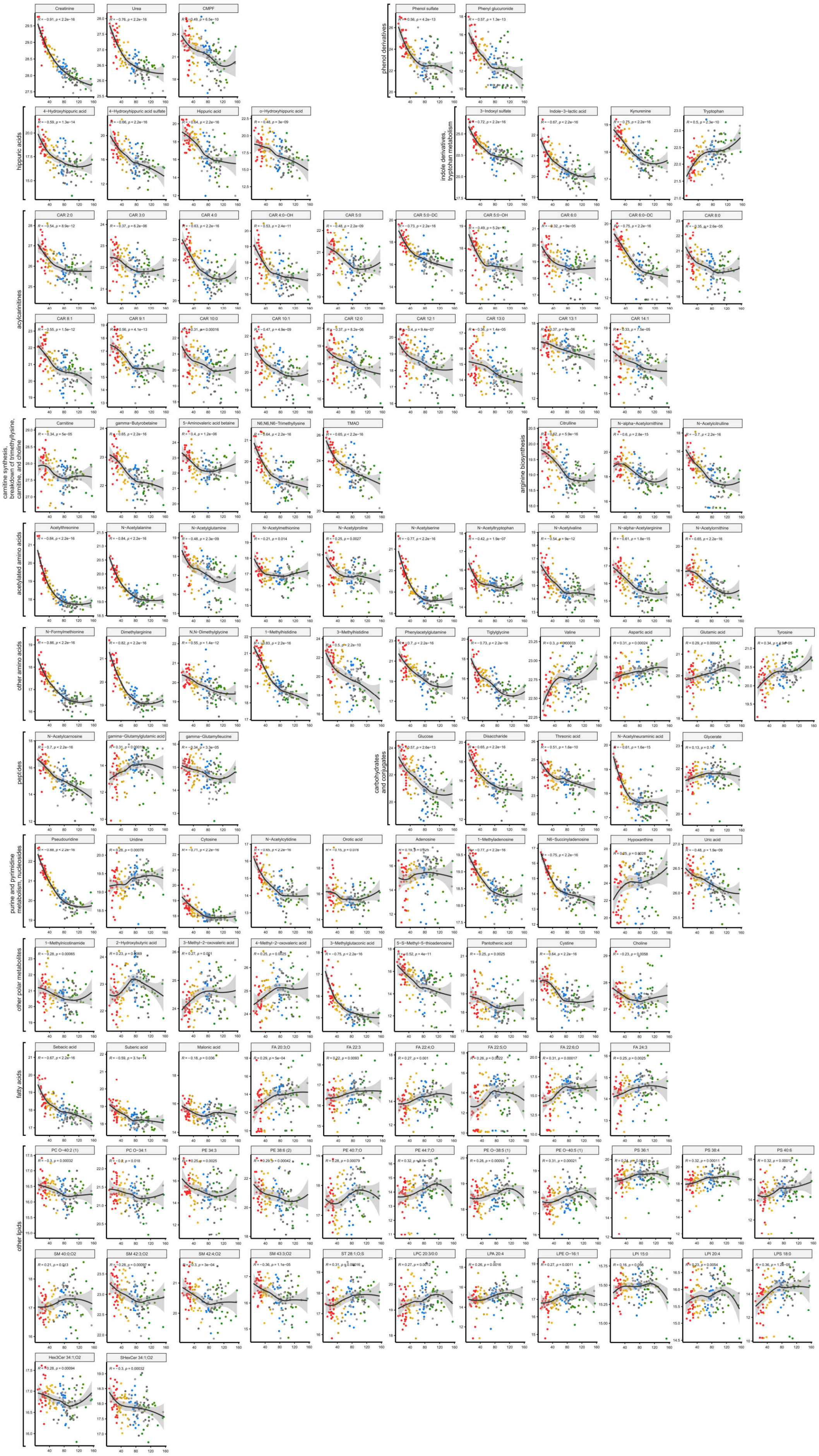

Supplemental Figure 7: Values measured in 121 metabolites (y-axis, arbitrary units) identified as significantly altered in stage 4 patients compared to controls are correlated with eGFR values (x-axis). The color of the dots corresponds to the stages of the kidney disease and the genotypes. The graphs are supplemented with loess curves and Spearman correlation coefficients with corresponding *P*-values (at the top of each panel).

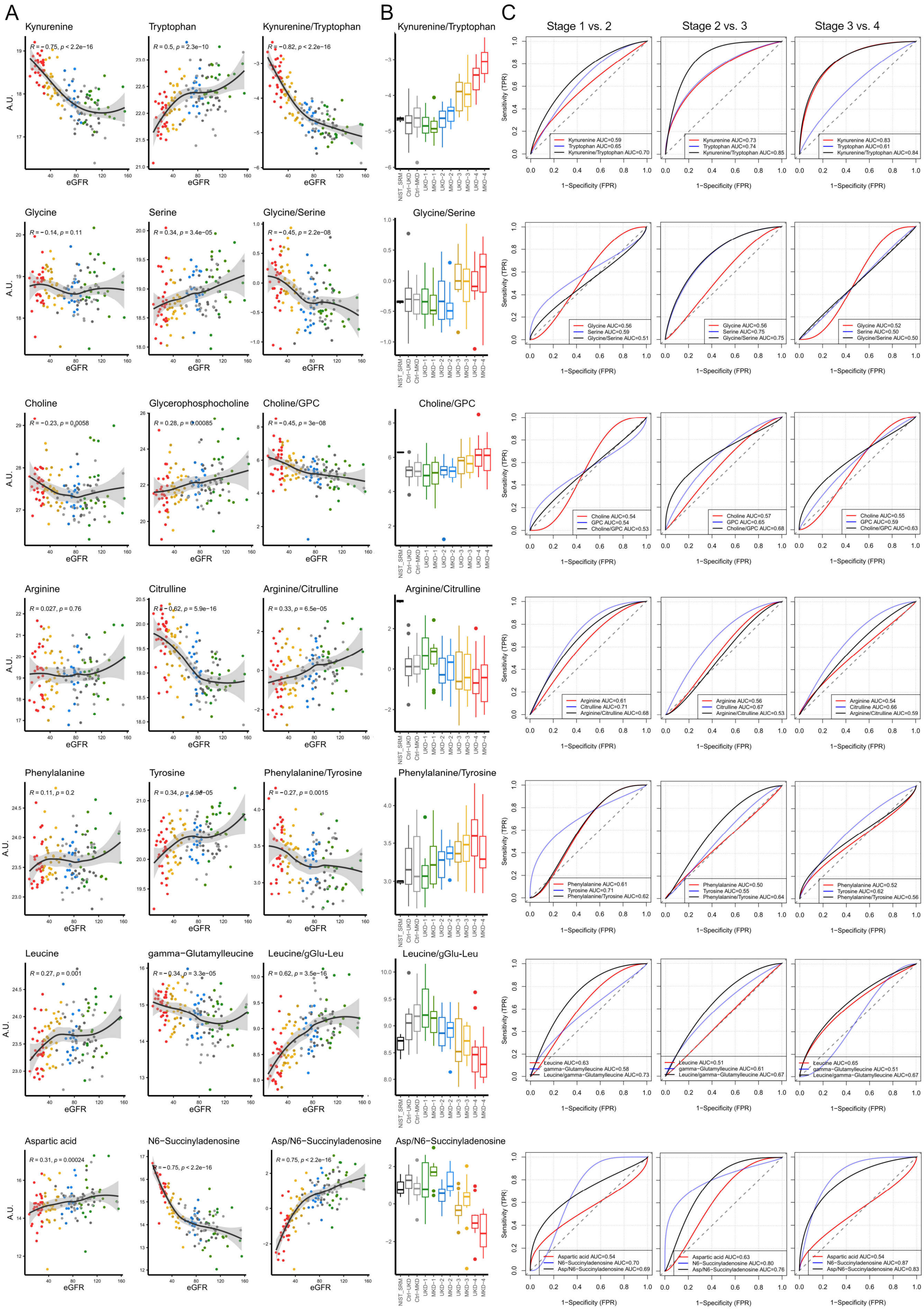

Supplemental Figure 8: Performance of selected metabolite ratios. (A) Correlation of measured values (y-axis, arbitrary units) and their ratios with eGFR values (on x-axis). The graphs are supplemented with loess curves and Spearman correlation coefficients with corresponding P-values (at the top of each panel). (B) The ratios of metabolite values displayed as boxplots. The color of the dots and boxplots (in a and b) corresponds to the stages of the kidney disease and the genotypes. (C) ROC analysis of the metabolites and their ratios. MKD, ADTKD-MUC1; UKD, ADTKD-UMOD; Ctrl-UKD, controls (genetically unaffected) from ADTKD-UMOD families; Ctrl-MKD, controls (genetically unaffected) from ADTKD-MUC1 families; NIST_SRM, NIST standard reference material.

Supplemental Table 1: Performance of the individual metabolites and their combinations in the random forest classification of ADTKD stages.

Metabolite	Classification errors:				out-of-bag error	accuracy
	**stage 1**	**stage 2**	**stage 3**	**stage 4**		
Creatinine	0.227	0.667	0.367	0.207	0.343	0.657
Creatinine + age	0.227	0.389	0.367	0.172	0.283	0.717
Creatinine + age + sex	0.182	0.611	0.133	0.207	0.273	0.727
Pseudouridine	0.318	0.556	0.300	0.207	0.323	0.677
N-Formylmethionine	0.318	0.778	0.433	0.345	0.444	0.556
Dimethylarginine	0.227	0.833	0.567	0.345	0.475	0.525
N-Acetylalanine	0.500	0.556	0.433	0.172	0.394	0.606
Acetylthreonine	0.636	0.778	0.500	0.345	0.535	0.465
P+F	0.273	0.500	0.317	0.241	0.323	0.677
P+D	0.273	0.500	0.367	0.241	0.338	0.662
P+AT	0.318	0.556	0.333	0.172	0.323	0.677
P+AA	0.227	0.444	0.400	0.259	0.323	0.677
P+F+D	0.136	0.472	0.333	0.207	0.283	0.717
P+F+D+AT	0.182	0.389	0.333	0.138	0.258	0.742
P+F+D+AT+AA	0.227	0.389	0.300	0.172	0.263	0.737
C+P	0.182	0.333	0.233	0.172	0.222	0.778
C+P+F	0.136	0.389	0.167	0.172	0.202	0.798
C+P+F+D	0.136	0.333	0.167	0.172	0.202	0.798
C+P+F+D+AT	0.136	0.278	0.233	0.172	0.202	0.798

C, creatinine; P, pseudouridine; F, N-Formylmethionine; D, Dimethylarginine; AA, N-Acetylalanine; AT, acetylthreonine.

Supplemental Table 2: The list of 121 significantly changed metabolites in comparison of ADTKD stage 4 vs. controls.

	**correlation with eGFR values (Spearman r)**	**log_2FC (ADTKD-*MUC1*-4/ctrl)**	**log_2FC (ADTKD-*UMOD*-4/Ctrl)**	**t-test ADTKD-4 vs. Ctrl (FDR adjusted *P*-value)**
Creatinine	-0.915	1.00	1.22	6.97E-23
Pseudouridine	-0.876	1.62	1.99	8.05E-24
N-Formylmethionine	-0.859	1.11	1.49	4.06E-21
N-Acetylalanine	-0.844	0.93	1.12	5.50E-22
Acetylthreonine	-0.840	1.49	2.16	2.22E-21
1-Methylhistidine	-0.826	2.13	2.11	8.05E-24
Dimethylarginine	-0.816	1.47	1.64	1.63E-24
1-Methyladenosine	-0.771	0.75	0.98	1.33E-15
N-Acetylserine	-0.767	1.33	1.68	2.26E-22
Urea	-0.761	1.04	1.06	6.47E-19
Kynurenine	-0.748	0.93	1.14	2.15E-16
CAR 6:0-DC	-0.747	3.04	3.58	1.10E-13
3-Methylglutaconic acid	-0.747	0.97	1.39	4.36E-12
N6-Succinyladenosine	-0.746	1.52	1.92	4.70E-20
Tiglylglycine	-0.731	1.81	2.93	4.91E-11
CAR 5:0-DC	-0.729	1.85	2.01	5.75E-14
3-Indoxyl sulfate	-0.722	1.68	2.88	4.91E-11
Cytosine	-0.709	0.85	0.95	3.20E-15
N-Acetylcarnosine	-0.699	1.56	1.68	2.40E-10
N-Acetylcitrulline	-0.697	2.33	3.18	7.03E-12
Phenylacetylglutamine	-0.697	1.70	2.35	1.31E-09
Indole-3-lactic acid	-0.675	0.79	1.30	7.38E-09
Sebacic acid	-0.674	0.83	1.45	1.71E-11
4-Hydroxyhippuric acid sulfate	-0.655	2.54	4.08	3.55E-09
N-Acetylornithine	-0.654	1.61	0.99	4.35E-07
Disaccharide	-0.654	2.07	2.58	4.50E-09
TMAO	-0.654	1.47	1.93	1.31E-09
N-Acetylcytidine	-0.651	1.02	1.68	2.01E-10
gamma-Butyrobetaine	-0.648	0.84	0.85	4.91E-11
N6,N6,N6-Trimethyllysine	-0.645	1.30	1.31	3.75E-13
Hippuric acid	-0.643	2.16	2.98	1.85E-09
Cystine	-0.635	0.90	0.95	1.23E-09
CAR 4:0	-0.629	1.48	1.34	1.51E-13
Citrulline	-0.615	0.85	0.76	5.36E-09
N-Acetylneuraminic acid	-0.608	0.89	1.30	1.29E-12
N-alpha-Acetylarginine	-0.607	1.21	1.25	4.36E-12
N-alpha-Acetylornithine	-0.604	1.26	0.68	2.55E-05
4-Hydroxyhippuric acid	-0.592	2.09	2.51	1.71E-11
Suberic acid	-0.585	0.61	0.85	1.17E-09
Phenyl glucuronide	-0.573	2.60	3.18	4.46E-09
Glucose	-0.568	1.47	1.83	3.61E-06
CAR 9:1	-0.564	1.61	1.66	3.48E-08
Phenol sulfate	-0.563	2.16	2.78	4.42E-10
N,N-Dimethylglycine	-0.553	0.78	0.84	1.87E-08
CAR 8:1	-0.553	1.55	1.26	1.48E-09
CAR 2:0	-0.536	0.78	1.10	4.32E-08
N-Acetylvaline	-0.536	1.25	1.60	3.79E-09
CAR 4:0-OH	-0.526	1.18	1.81	1.52E-09
5-S-Methyl-5-thioadenosine	-0.521	1.62	1.86	1.19E-06
Threonic acid	-0.507	0.54	0.67	1.03E-04
3-Methylhistidine	-0.504	2.26	2.42	3.25E-08
CAR 5:0-OH	-0.495	1.12	0.99	2.19E-09
CMPF	-0.492	2.17	2.76	9.17E-05
Uric acid	-0.480	0.11	0.47	1.82E-04
CAR 5:0	-0.479	0.89	0.75	5.10E-07
N-Acetylglutamine	-0.478	0.67	1.25	8.21E-05
o-Hydroxyhippuric acid	-0.475	1.49	2.26	2.67E-04
CAR 10:1	-0.469	0.94	1.33	8.36E-07
N-Acetyltryptophan	-0.423	0.49	0.95	5.18E-06

CAR 12:1	-0.400	0.69	1.40	4.49E-04
5-Aminovaleric acid betaine	-0.397	0.92	0.95	1.14E-08
CAR 3:0	-0.372	0.81	0.40	4.40E-04
CAR 13:1	-0.367	0.74	1.13	5.46E-04
CAR 12:0	-0.367	0.50	1.24	9.66E-04
SM 43:3;O2	-0.361	0.51	0.44	9.69E-04
CAR 13:0	-0.358	0.73	1.08	5.97E-04
CAR 8:0	-0.347	0.76	1.44	2.70E-04
gamma-Glutamylleucine	-0.343	0.44	0.48	2.72E-03
Carnitine	-0.336	0.55	0.26	1.72E-04
CAR 14:1	-0.328	0.45	1.15	1.12E-02
CAR 6:0	-0.325	0.62	1.22	4.86E-04
CAR 10:0	-0.313	0.64	1.47	9.90E-04
SM 42:4;O2	-0.301	0.34	0.34	1.68E-02
SHexCer 34:1;O2	-0.300	0.26	0.31	4.79E-02
PC O-40:2 (1)	-0.300	0.29	0.31	2.68E-03
PE 38:6 (2)	-0.294	0.65	0.89	3.88E-02
1-Methylnicotinamide	-0.285	0.51	0.78	8.15E-03
Hex3Cer 34:1;O2	-0.277	0.27	0.28	1.91E-03
SM 42:3;O2	-0.276	0.27	0.30	1.93E-02
PE 34:3	-0.254	0.98	1.13	1.98E-02
Pantothenic acid	-0.253	0.62	0.60	1.23E-03
N-Acetylproline	-0.251	0.22	0.49	4.35E-03
Choline	-0.232	0.26	0.37	3.27E-02
N-Acetylmethionine	-0.208	0.27	0.69	1.68E-02
PC O-34:1	-0.200	0.26	0.25	3.02E-02
Malonic acid	-0.177	0.31	0.38	3.33E-02
Orotic acid	-0.149	0.94	0.26	2.04E-02
Glycerate	0.126	-0.36	-0.26	2.13E-02
LPI 15:0	0.162	-0.11	-0.08	2.20E-02
Adenosine	0.190	-1.48	-1.01	4.99E-04
SM 40:0;O2	0.210	-0.37	-0.29	3.58E-02
FA 22:3	0.219	-0.35	-0.36	2.27E-02
2-Hydroxybutyric acid	0.227	-0.59	-0.35	7.27E-03
LPI 20:4	0.234	-0.39	-0.23	1.66E-02
PS 36:1	0.239	-1.08	-0.87	1.43E-03
Hypoxanthine	0.251	-1.93	-1.67	7.39E-03
FA 24:3	0.253	-0.42	-0.51	1.04E-02
4-Methyl-2-oxovaleric acid	0.253	-0.52	-0.56	5.24E-03
FA 22:5;O	0.257	-1.53	-1.48	5.81E-03
LPA 20:4	0.264	-0.96	-0.50	2.69E-02
LPC 20:3/0:0	0.271	-0.40	-0.39	2.40E-02
LPE O-16:1	0.273	-0.62	-0.24	4.37E-02
FA 22:4;O	0.274	-0.89	-0.65	1.34E-02
3-Methyl-2-oxovaleric acid	0.275	-0.54	-0.34	4.35E-03
PE O-38:5 (1)	0.277	-0.40	-0.30	8.15E-03
PE 40:7;O	0.281	-0.52	-0.34	5.57E-03
Uridine	0.281	-0.20	-0.24	3.63E-02
FA 20:3;O	0.290	-1.38	-1.17	2.04E-02
Glutamic acid	0.294	-0.38	-0.38	1.12E-02
Valine	0.299	-0.09	-0.23	1.20E-02
Aspartic acid	0.305	-0.37	-0.52	4.46E-02
PE O-40:5 (1)	0.309	-0.58	-0.48	4.86E-04
gamma-Glutamylglutamic acid	0.310	-0.68	-1.25	4.86E-04
FA 22:6;O	0.313	-1.95	-1.68	1.89E-03
ST 28:1;O;S	0.314	-0.77	-0.41	4.79E-03
PS 40:6	0.319	-1.29	-0.86	1.38E-03
PS 38:4	0.321	-1.27	-0.92	1.65E-05
PE 44:7;O	0.323	-1.09	-0.82	4.49E-04
Tyrosine	0.336	-0.21	-0.29	2.12E-02
LPS 18:0	0.361	-1.59	-1.00	2.62E-04
Tryptophan	0.503	-0.23	-0.51	1.18E-03

Supplemental Methods

Sample preparation

LC-MS-grade solvents, including acetonitrile, isopropanol, methanol, water, and methyl *tert*-butyl ether (MTBE), as well as mobile phase modifiers like ammonium formate, ammonium acetate, formic acid, and acetic acid, were sourced from J.T. Baker, Merck, and VWR International (Prague, Czech Republic). Internal standards were acquired from Cambridge Isotope Laboratories (Tewksbury, MA, USA), Cayman Chemical (Tallinn, Estonia) and the Merck Standard Reference Material 1950 Metabolites in Frozen Human Plasma (NIST SRM 1950) was obtained from Merck.

A 25 µL aliquot of plasma was mixed with 765 µL of an ice-cold methanol/MTBE solution (165 µL methanol and 600 µL MTBE) containing internal standards [1]. The mixture was shaken for 30 s. Next, 165 µL of 10% methanol, also containing internal standards [1], was added, vortexed for 10 s, and centrifuged at 16,000 rpm for 5 min at 4°C.

A 70 µL aliquot of the bottom phase was collected and evaporated for metabolomic analysis. The dried serum extracts were resuspended in 70 µL of an acetonitrile/water (4:1) mixture containing two internal standards—12-[[(cyclohexylamino)carbonyl]amino]-dodecanoic acid (CUDA) and Val-Tyr-Val—shaken for 30 s, centrifuged at 16,000 rpm for 5 min at 4°C, and analyzed using hydrophilic interaction chromatography (HILIC) metabolomics platform. Another 70 µL aliquot of the bottom phase was mixed with 210 µL of an isopropanol/acetonitrile (1:1) solution, shaken for 30 s, centrifuged at 16,000 rpm for 5 min at 4°C, and the supernatant was evaporated. The dried extracts were resuspended in 5% methanol/0.2% formic acid with CUDA and Val-Tyr-Val as internal standards, shaken for 30 s, centrifuged at 16,000 rpm for 5 min at 4°C, and analyzed using the reversed-phase liquid chromatography (RPLC) metabolomics platform.

For lipidomic analysis, 200 µL of the upper phase was collected, evaporated, and the dried extracts were resuspended in 100 µL methanol containing the internal standard CUDA, shaken for 30 s, centrifuged at 16,000 rpm for 5 min at 4°C, and prepared for LC-MS analysis.

LC-MS/MS analysis

The LC-MS system consisted of a Vanquish UHPLC system (Thermo Fisher Scientific, Bremen, Germany) with a heated electrospray ionization (HESI-II) probe and a Q Exactive Plus mass spectrometer (Thermo Fisher Scientific). Several measures were implemented to ensure quality control: (i) randomization of samples within the sequence, (ii) regular injection of quality control (QC) pool samples at the beginning, end, and after every ten actual samples for each matrix, (iii) analysis of method blanks, (iv) analysis of serial dilution samples prepared from the QC sample (0, 1/16, 1/8, 1/4, 1/2, 1), and (v) monitoring of chromatographic peak shape, retention time, and internal standard intensity [1].

For polar metabolite separation based on the HILIC mechanism, an ACQUITY Premier BEH Amide column (50 mm × 2.1 mm i.d.; 1.7 µm particle size) equipped with a VanGuard FIT cartridge (5 mm × 2.1 mm i.d.; 1.7 µm particle size) (Waters, Milford, MA, USA) was used. The separation was performed at a flow rate of 0.4 mL/min, with the column maintained at 45°C. The mobile phase consisted of (A) water containing 10 mM ammonium formate and 0.125% formic acid, and (B) acetonitrile/water (95:5) with 10 mM ammonium formate and 0.125% formic acid. The gradient was as follows: 0 min 100% (B); 0–1 min 100% (B); 1–3.9 min from 100% to 70% (B); 3.9–5.1 min from 70% to 30% (B); 5.1–6.4 min from 30% to 100% (B); 6.4–7.5 min 100% (B) +1 min preinjection steps. Injection volumes were 1 µL for ES(+). The sample temperature was maintained at 4°C.

For separation of polar metabolites using the RPLC mechanism, an ACQUITY Premier HSS T3 column (50 mm × 2.1 mm i.d.; 1.8 µm particle size) with a VanGuard FIT cartridge (5 mm × 2.1 mm i.d.; 1.8 µm particle size) (Waters) was employed. The column was maintained at 45°C. The mobile phase consisted of (A) water with 0.2% formic acid, and (B) methanol with 0.1% formic acid. The gradient was: 0 min 1% (B) 0.3 mL/min; 0–0.5 min 1% (B) 0.3 mL/min; 0.5–2 min from 1% to 60% (B) 0.3 mL/min; 2–2.3 min from 60% to 95% (B) from 0.3 mL/min to 0.5 mL/min; 2.3–3.0 min 95% (B) 0.5 mL/min; 3.0–3.1 min from 95% to 1% (B) 0.5 mL/min; 3.1–4 min 1% (B) 0.5 mL/min; 4–4.1 min 1% (B) from 0.5 mL/min to 0.3 mL/min; 4.1–4.5 min 1% (B) 0.3 mL/min + 1 min preinjection steps. An injection volume of 5 µL was used for ESI(–), and the sample temperature was kept at 4°C.

The ion source parameters were: sheath gas pressure at 50 arbitrary units, auxiliary gas flow at 13 units, sweep gas flow at 3 units, capillary temperature of 300°C, auxiliary gas heater temperature of 370°C, and spray voltage of 3.6 kV for ESI(+), −3.0 kV for ESI(−). The mass spectrometer was set to an MS1 mass range of m/z 60–900, with an MS1 resolving power of 35,000 FWHM (m/z 200), and 3 data-dependent scans per cycle. MS/MS resolving power was set to 17,500 FWHM (m/z 200), and normalized collision energies of 20%, 30%, and 40% were applied for both polarities.

For complex lipid separation using the RPLC mechanism, the ACQUITY Premier BEH C18 column (50 mm × 2.1 mm i.d.; 1.7 µm particle size) with a VanGuard FIT cartridge (5 mm × 2.1 mm i.d.; 1.7 µm particle size) (Waters) was used. The flow rate

was 0.6 mL/min, with the column kept at 65°C. For LC-ESI(+)-MS lipidomic analysis, the mobile phase comprised (A) acetonitrile/water (60:40) with 10 mM ammonium formate and 0.1% formic acid, and (B) isopropanol/acetonitrile/water (90:10:0.1) with 10 mM ammonium formate and 0.1% formic acid. For LC-ESI(−)-MS, mobile phase (A) contained acetonitrile/water (60:40) with 10 mM ammonium acetate and 0.1% acetic acid, and mobile phase (B) comprised isopropanol/acetonitrile/water (90:10:0.1) with 10 mM ammonium acetate and 0.1% acetic acid. The gradient for LC-ESI(+)-MS was: 0 min 15% (B); 0–1 min from 15% to 30% (B); 1–1.3 min from 30% to 48% (B); 1.3–5.5 min from 48% to 82% (B); 5.5–5.8 min from 82% to 99% (B); 5.8–6 min 99% (B); 6–6.1 min from 99% to 15% (B); 6.1–7 min 15% (B) +1 min preinjection steps. The LC-ESI(−)-MS gradient was similar, with slight modifications: 0 min 15% (B); 0–1 min from 15% to 30% (B); 1–1.3 min from 30% to 48% (B); 1.3–4.8 min from 48% to 76% (B); 4.8–4.9 min from 76% to 99% (B); 4.9–5.3 min 99% (B); 5.3–5.4 min from 99% to 15% (B); 5.4–6.3 min 15% (B) +1 min preinjection steps. Injection volumes were 0.5 µL for ESI(+), and 5 µL for ESI(−). The sample temperature was kept at 4°C.

The ion source parameters were: sheath gas pressure at 60 units, auxiliary gas flow at 25 units, sweep gas flow at 2 units, capillary temperature of 300°C, auxiliary gas heater temperature of 370°C, and spray voltage of 3.6 kV for ESI(+), −3.0 kV for ESI(−). The mass spectrometer settings were: MS1 mass range of m/z 200–1700, MS1 resolving power of 35,000 FWHM (m/z 200), and 3 data-dependent scans per cycle. For MS/MS experiments, a normalized collision energy of 20% was applied in positive ion mode, while 10%, 20%, and 30% energies were set for negative ion mode.

Data processing

LC-MS instrumental files generated from metabolomic and lipidomic analyses were processed using MS-DIAL v. 4.9.221218 software [2] with the following parameters: (i) data collection: MS1 tolerance of 0.01; MS2 tolerance of 0.025; (ii) peak detection: minimum peak height of 15,000, mass slice width of 0.05, smoothing method as Linear Weighted Moving Average, and smoothing level set to 2; (iii) MS/MS identification settings: MS1 accurate mass tolerance of 0.005, MS2 accurate mass tolerance of 0.005, identification score cut-offs of 80%; (iv) alignment: retention time tolerance of 0.05 min, MS1 tolerance of 0.01 Da, peak count filter of 5%, and gap filling by compulsion.

Polar metabolites were annotated using retention time–*m/z* matches from an in-house spectral library along with MS/MS libraries such as NIST20, MassBank.us, and MS-DIAL MS/MS library v. 15. Complex lipids were annotated with in silico MS/MS spectra provided in MS-DIAL software.

The exported datasets for each platform, expressed as signal intensity from the detector (peak heights), were filtered by removing metabolites that met the following criteria: (i) a max sample peak height/blank peak height average ratio of less than 10, (ii) an $R^2 < 0.8$ in a QC sample dilution series, and (iii) a relative standard deviation (RSD) greater than 30% in QC samples injected between every ten actual study samples. Data normalization was performed using locally estimated scatterplot smoothing (LOESS) based on QC samples [1].

Missing values for individual metabolites were imputed by assigning each missing observation a value equal to one-fifth of that metabolite’s lowest measured intensity.

Statistical analyses

Statistical analyses of log2-transformed data were performed using R, a language and environment for statistical computing. Unpaired Student´s t-test and ANOVA were used to compare continuous variables and chi-square or Fisher´s exact test were used for categorical variables. Multiple comparisons were accounted for by computing the false discovery rate (FDR) adjusted P-values. Unless otherwise stated, an FDR threshold of 0.05 was used to consider the results significant. Multivariate analysis (PCA, OPLS) was performed using the “prcomp” function and “ropls” package to examine data variation. The robustness of the created OPLS models was evaluated by monitoring the fitness of the model (R2) and predictive ability (Q2). R2Y and Q2 values closer to 1 indicate that the model is more stable and reliable. The models were validated using a permutation test (20 permutations). All R2 and Q2 values were smaller than the values in the actual model, indicating that there was no overfitting in the OPLS model. The contribution rate of a variable is described by the variable importance of the projection (VIP) value. The performance of metabolites on differentiating disease stages was evaluated using ROC analysis and a random forest algorithm using the “randomForest” package. Pathway analysis was performed in MetaboAnalyst version 6.0 [3].

References

[1] J. Hricko, L. Rudl Kulhava, M. Paucova, M. Novakova, O. Kuda, O. Fiehn, T. Cajka: Short-term stability of serum and liver extracts for untargeted metabolomics and lipidomics. Antioxidants 12 (2023) 986. (doi: 10.3390/antiox12050986)
[2] H. Tsugawa, K. Ikeda, M. Takahashi, A. Satoh, Y. Mori, H. Uchino, N. Okahashi, Y. Yamada, I. Tada, P. Bonini, Y. Higashi, Y. Okazaki, Z. Zhou, Z.-J. Zhu, J. Koelmel, T. Cajka, O. Fiehn, K. Saito, M. Arita, M. Arita: A lipidome atlas in MS-DIAL 4. Nature Biotechnology 38 (2020) 1159–1163. (doi: 10.1038/s41587-020-0531-2)
[3] Z. Pang Z, Y. Lu, G. Zhou, F. Hui, L. Xu, C. Viau, A.F. Spigelman, P.E. MacDonald, D.S. Wishart, S. Li, J. Xia: MetaboAnalyst 6.0: towards a unified platform for metabolomics data processing, analysis and interpretation. Nucleic Acids Res 52 (2024) W398-W406. (doi:10.1093/nar/gkae253)
